# Identification and external validation of a prognostic signature based on myeloid-derived suppressor cell-related lncRNAs for hepatocellular carcinoma

**DOI:** 10.1186/s41065-026-00664-z

**Published:** 2026-03-19

**Authors:** Tong Mu, Shunyao Zhang, Yun Zhong, Zijian Zhou, Lei Xie, Yajie Zhou, Wenxiong Zhang, Liang Zhai

**Affiliations:** 1 Department of Thoracic Surgery, The Second Affiliated Hospital, Jiangxi Medical College, Nanchang University, No.1 Minde Road, Nanchang, 330006 China; 2 The Second Clinical Medical School, Jiangxi Medical College, Nanchang University, Nanchang, 330088 China; 3 Department of Hepatobiliary Surgery, The Second Affiliated Hospital, Jiangxi Medical College, Nanchang University, Nanchang, 330006 China; 4 Operating Room, The Second Affiliated Hospital, Jiangxi Medical College, Nanchang University, 1 Minde Road, Donghu District, Nanchang, 330006 China

**Keywords:** Myeloid-derived suppressor cell, LncRNAs, Hepatocellular carcinoma, Prognostic signature, Immunotherapy

## Abstract

**Background:**

Hepatocellular carcinoma (HCC) outcomes remain suboptimal. Myeloid-derived suppressor cells (MDSCs) exhibit notable immunosuppressive and pro-tumorigenic properties. However, the relationship with HCC remains insufficiently explored.

**Methods:**

Using TCGA data, we developed an MDSC-associated lncRNA prognostic model and a clinical nomogram. To explore the model’s mechanistic basis and clinical significance, enrichment analysis, tumor mutation burden (TMB) analysis, tumor microenvironment (TME) evaluation, immunotherapy response prediction, and drug sensitivity assessment were performed. RT-qPCR was utilized to confirm lncRNA expression.

**Results:**

A 7-lncRNA prognostic signature was established. High-risk patients exhibited significantly worse survival (*p* < 0.001). The nomogram demonstrated superior accuracy over models excluding the risk evaluation. Enrichment analysis exposed that metabolic and immune-associated pathways were predominant within low-risk cohort, while cell proliferation and gene expression regulation dominant across the high-risk population. Meanwhile, an increased TMB and a degraded TME appeared in the high-risk population. The drug responsiveness evaluation revealed that sorafenib, axitinib, and others exhibited enhanced effectiveness among the low-risk population. High-risk individuals displayed enhanced reactions to medications like staurosporine, savolitinib, and others.

**Conclusions:**

The prognostic model constructed based on the seven MDSCs-associated lncRNAs showed good application value in assessing prognosis and guiding clinical therapy.

**Supplementary Information:**

The online version contains supplementary material available at 10.1186/s41065-026-00664-z.

## Introduction

Liver cancer remains a critical global health challenge and is one of the leading causes of cancer-related death worldwide [1]. Hepatocellular carcinoma (HCC) is the predominant histological subtype, accounting for approximately 75% to 85% of primary liver cancers [2], and the overall 5-year survival rate remains unsatisfactory [1]. Although TNM staging is widely used in clinical practice, its ability to predict long-term outcomes is limited because it cannot fully reflect tumor heterogeneity or the influence of the underlying liver condition [3]. Recent evidence indicates that chromosomal rearrangements such as translocations can produce previously unrecognized fusion genes, highlighting the complexity of cancer genomes [4]. Consequently, increasing attention has been directed toward molecular biomarkers to provide a more refined understanding of HCC biology. Biomarkers such as PRSS35 [5] and des-gamma-carboxy prothrombin (DCP) [6] have shown promise in diagnosis and patient stratification, but the limited sensitivity and specificity of single biomarkers reduce their reliability in clinical application. To improve prognostic precision, integrated predictive models combining molecular features with clinical parameters have been proposed, including the TMB-related prognostic model reported by Tang et al. [7].

At the same time, the therapeutic landscape of HCC has evolved rapidly, and immunotherapy has become an important treatment modality for advanced disease. However, its clinical benefit remains heterogeneous, partly because HCC usually develops in a chronically inflamed and immunosuppressive hepatic microenvironment; in addition, important concerns remain in specific clinical settings such as liver transplantation [8]. Myeloid-derived suppressor cells (MDSCs), which originate in the bone marrow, are a heterogeneous population of immature myeloid cells with potent immunosuppressive activity and are recognized as key contributors to tumor immune escape [9]. In HCC, MDSCs facilitate tumor progression by inhibiting antitumor immune responses and reducing effective immune-cell infiltration within the tumor microenvironment. Long noncoding RNAs (lncRNAs), a class of non-coding transcripts longer than 200 nucleotides, also play crucial roles in tumor progression, metastasis, therapeutic resistance, and immune regulation [10]. Increasing evidence suggests that lncRNAs can modulate both the expansion and immunosuppressive activity of MDSCs, thereby contributing to tumor immune escape and remodeling of the HCC immune microenvironment [11].

Recent studies have further highlighted the importance of biologically informed biomarkers in HCC. Higher expression of Vav1 has been reported to be associated with unfavorable clinicopathological features and poor prognosis in HCC, supporting the relevance of immune- and signaling-related molecules in disease progression [12]. PRDX6 has also been implicated in HCC progression and prognosis, with experimental evidence suggesting that it may promote malignant phenotypes in HCC cells [13]. In addition, integrative transcriptomic analysis has identified CDT1 as a potential therapeutic target in the progression from non-alcoholic fatty liver disease to HCC, further underscoring the value of computational biomarker discovery and model development in this malignancy [14]. These findings indicate that HCC prognostic research is increasingly moving toward mechanism-based biomarkers that better capture both tumor biology and the immune microenvironment.

Studies in recent years have already shown the prognostic value of immune-related lncRNA signatures in cancer; for example, Peng et al. developed a risk signature for ovarian cancer using immune-related lncRNAs [15]. However, an MDSC-related lncRNA-based prognostic model specifically for HCC has not yet been fully established. Therefore, this study aimed to construct and externally validate a prognostic signature based on MDSC-associated lncRNAs for HCC. Furthermore, the biological basis and clinical relevance of this model were explored through functional enrichment analysis, tumor mutation burden (TMB) evaluation, tumor microenvironment (TME) characterization, immunotherapy response prediction, and drug sensitivity assessment.

## Materials and methods

### Data acquisition and accessibility

This study primarily utilized data from the University of California, Santa Cruz (UCSC) Xena database [16]. The Cancer Genome Atlas (TCGA) database within Xena served to extract transcriptomic data from 372 HCC cases and clinical information from 376 patients. Moreover, somatic mutation data of tumor cells were obtained via the TCGA repository (https://portal.gdc.cancer.gov/repository, accessed up to Jun 10, 2025). Transcriptome data were obtained in the form of STAR-Counts for further analysis. To refine the dataset, we employed Perl (version 5.30.0.1) to filter patients with complete clinical and transcriptional data, ensuring their inclusion in both datasets.

At the study design stage, a precision-based approach was applied to estimate the required sample size. The goal was to ensure that the 95% confidence interval (CI) half-width for the estimated overall survival rate would not exceed ± 5%, with a two-sided significance level of α = 0.05. Based on previous reports in hepatocellular carcinoma cohorts, the expected event proportion during follow-up was set at *p* = 0.35. Accordingly, the required sample size was calculated using the formula: N = (Z² × p × (1 − p)) / w², where *Z* is the standard normal quantile corresponding to α/2 (Z = 1.96), *p* is the expected event proportion (0.35), and w is the allowable half-width of the CI (0.05). Substituting these values yields *N* ≈ 350, our study included 369 patients, the sample size meets the theoretical requirement and is sufficient to ensure adequate precision in estimating the overall survival rate.

### Select MDSCs-associated genes lncRNAs

We searched GeneCards platform(https://www.genecards.org/, last accessed July 22, 2025) using the term “myeloid-derived suppressor cell” and retained protein-coding genes with a relevance score > 20. To further improve specificity and maintain a manageable candidate set for downstream curation, only the top 200 ranked genes were kept as preliminary candidates. These genes were then manually reviewed through the literature, and only genes with experimental or strong literature-supported evidence of causal or significant associations with MDSC biology were finally included.

To identify lncRNAs associated with MDSCs, we performed a Pearson correlation test to evaluate their relationships with MDSCs-related genes, applying a selection criterion of |correlation coefficient| > 0.4 and p-value < 0.05. Chosen lncRNAs were assessed for differential expressions with the “DESeq2” package, keeping those that met the significance criteria of an adjusted p-value < 0.05 and |log2FC| > 1. To develop and validate the model, all patients were randomly divided into two cohorts of equal size: a training cohort for model construction with a test segment for verification. Cox regression analysis of a single variable was conducted on the training cohort to identify lncRNAs associated with MDSCs that influence patient survival. To prevent overfitting, we further applied the LASSO regression. Next, researchers applied multivariate Cox regression methods to select vital lncRNAs linked with MDSCs, developing a risk evaluation framework.

### Prognostic framework of lncRNAs tied to MDSCs

We applied findings from developing a forecast system. The equation used to develop this predictive framework is given by: risk score = ∑expression (lncRNAs) * coefficient (lncRNAs). Every patient was allocated in low- or high-risk sets by median risk threshold.

To examine overall survival (OS) disparities across these sets, we executed Kaplan-Meier (K-M) survival evaluation in all three populations. The system’s accuracy in forecasting outcomes was analyzed using ROC graphs for survival projections at one, three, and five years. The analysis using “survival” and “survminer” in R.

Additionally, our team utilized PCA to evaluate the system’s categorization accuracy through assessing gene expression trends in MDSCs-linked genes, associated lncRNAs, risk-related lncRNAs, and the entire gene set. Furthermore, univariate alongside multivariate Cox analyses assessed the model’s specific prognostic strength with clinical aspects. Lastly, K-M survival analysis was applied across different clinical subgroups to analysis the robustness and feasibility for diverse patient characteristics.

To further examine the reproducibility of tumor-associated expression patterns in independent datasets, three GEO cohorts (GSE138178, GSE148355, and GSE214846) containing HCC and non-tumor/normal liver samples were used for external validation. For each lncRNA, expression differences between tumor and non-tumor/normal samples were compared using the Wilcoxon rank-sum test in R.

### Creation of a nomogram to predict patient prognosis occurred

“RMS” was employed to develop a diagram incorporating risk classification, age, gender, grade, Child Pugh Grade, HBV or HCV status and tumor stage as predictive variables for estimating patient of HCC prognostic status at 1, 3, and 5 years [17]. Additionally, the system’s effectiveness was assessed to determine its value in forecasting prognosis.

### Pathway and functional annotation analysis

We applied the filtering criteria of |log2 fold change| > 1 and both p-value < 0.05 to identify genes with differential expression between the risk cohorts. The functional enrichment of these genes was next examined with the Gene Ontology (GO) and Kyoto Encyclopedia of Genes and Genomes (KEGG) databases, employing the “clusterProfiler” for analysis.

Subsequently, we performed gene set enrichment analysis (GSEA) to retrieve data files (in gmt format) through GSEA website (www.gsea-msigdb.org). Differences in pathway enrichment between risk groups were identified by p-value < 0.05.

### Impact of tumor mutational burden on prognosis

Tumor somatic mutation information from TCGA was processed and examined by the “TCGAbiolinks” package. Then we select “maftools” to generate the waterfall plots. In addition, we used TMB score that we calculated and risk score of the risk signature to analysis HCC patients’ survival [18, 19].

### Evaluations of tumor microenvironment and immuno-infiltration

Our team then employed the “ESTIMATE” tool to examine changes in stromal, immune, and ESTIMATE scores, plus tumor purity levels, across distinct group pairs. Additionally, further insights were gathered from the Tumor Immune Estimation Resource (TIMER) 2.0 platform (http://timer.cistrome.org/, accessed until July 30, 2025). Several computational methods, including “TIMER”, “CIBERSORT”, “CIBERSORT-ABS”, “QUANTISEQ”, “XCELL”, “EPIC”, and “MCPCOUNTER” were applied to refine the correlation between specific immune cell populations and risk scores [20]. To comprehensively analyze immune cell composition, an immune infiltration assessment was conducted across 22 distinct immune cell lineages.

To further evaluate the association between each signature lncRNA and MDSC infiltration, we performed linear regression (and correlation) analyses between lncRNA expression levels and the MDSC score across the TCGA-HCC cohort, and visualized the relationships using scatter plots.

### Drug sensitivity prediction

To comprehensively analyze tumor immune differences across HCC risk groups, the Tumor Immune Dysfunction and Exclusion (TIDE) database (http://tide.dfci.harvard.edu/, accessed until July 11, 2025) was utilized to obtain TIDE scores and associated information for HCC individuals [21]. Furthermore, we retrieved the immune checkpoint expression data for PD-1 and CTLA-4 in HCC patients from TCIA (https://tcia.at/, until July 11, 2025) for further research.

Based on this information, single-sample gene set enrichment analysis (ssGSEA) was performed utilizing the “GSVA” R package to explore immune-related differences. Furthermore, we downloaded “oncoPredict” in R to gauge half-maximal inhibitory concentrations (IC50) for widely used cancer therapies [22]. IC50 values underwent statistical evaluation across cohorts via the Wilcoxon rank-sum test.

### Subcellular localization analysis

The subcellular localization of the different prognostic lncRNAs was investigated using the lncATLAS (https://lncatlas.crg.eu/) and RNALocate (http://rnalocate.org/)databases. For lncRNAs available in lncATLAS, localization patterns across multiple human cell lines were evaluated based on the cytoplasmic/nuclear relative concentration index (CN-RCI), where RCI < 0 indicates nuclear enrichment and RCI > 0 indicates cytoplasmic enrichment. The lncRNAs not comprehensively annotated in lncATLAS, subcellular localization information was retrieved from RNALocate. Based on these databases, each lncRNA was categorized as predominantly cytoplasmic, nuclear, or distributed across multiple cellular compartments.

### RT-qPCR Validation

The normal liver epithelial cell line (LO2), alongside HCC cell lines (HepG2 and Huh7) was obtained from the Cell Bank of the Typical Culture Preservation Commission, affiliated with the Chinese Academy of Medical Sciences in Shanghai, China. Cultivation of LO2 cells occurred in RPMI-1640 medium supplemented with 10% fetal bovine serum, while HepG2 and Huh7 cells were grown in DMEM medium. Extraction of total RNA were carried out with Trizol reagent (Sangon Biotech, Shanghai, China). Subsequently, cDNA was synthesized with a kit provided by Accurate Biology (Hunan, China), and RT-qPCR analysis ensued using the SYBR Green premixed qPCR kit (Thermo Fisher Scientific, Waltham, USA) on a Roche LightCycler 480 II instrument (Roche, Basel, Switzerland). The expression levels of genes were determined with precision through the 2^−ΔΔCt approach, with the detailed sequences comprehensively documented in Table S1.

Furthermore, protein-level expression of the genes in both normal and HCC tissues was assessed by referencing the Human Protein Atlas database (https://www.proteinatlas.org/).

## Results

### Screening MDSCs-associated lncRNAs

**Fig. 1:**
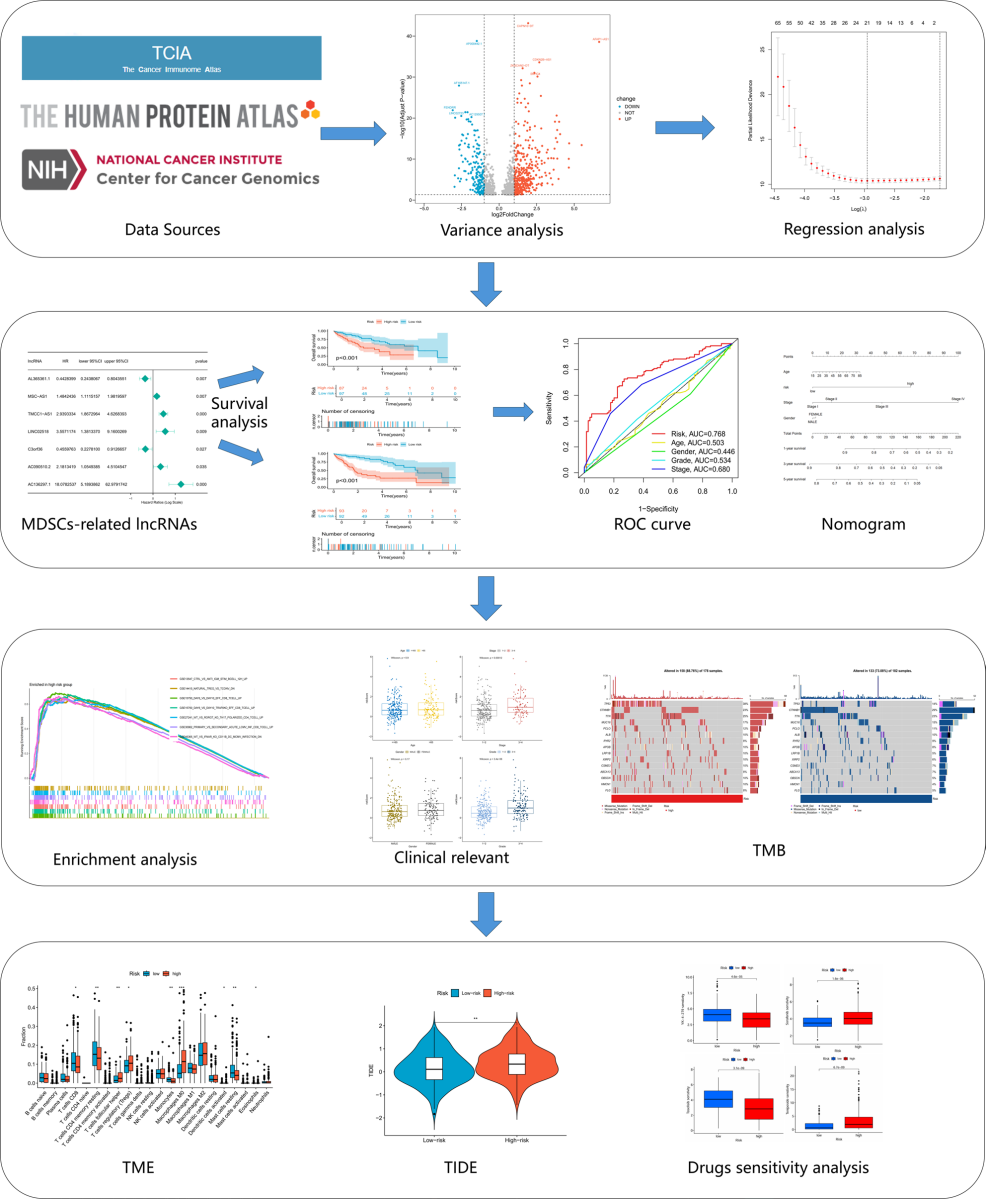
Flow Chart

**Fig. 2:**
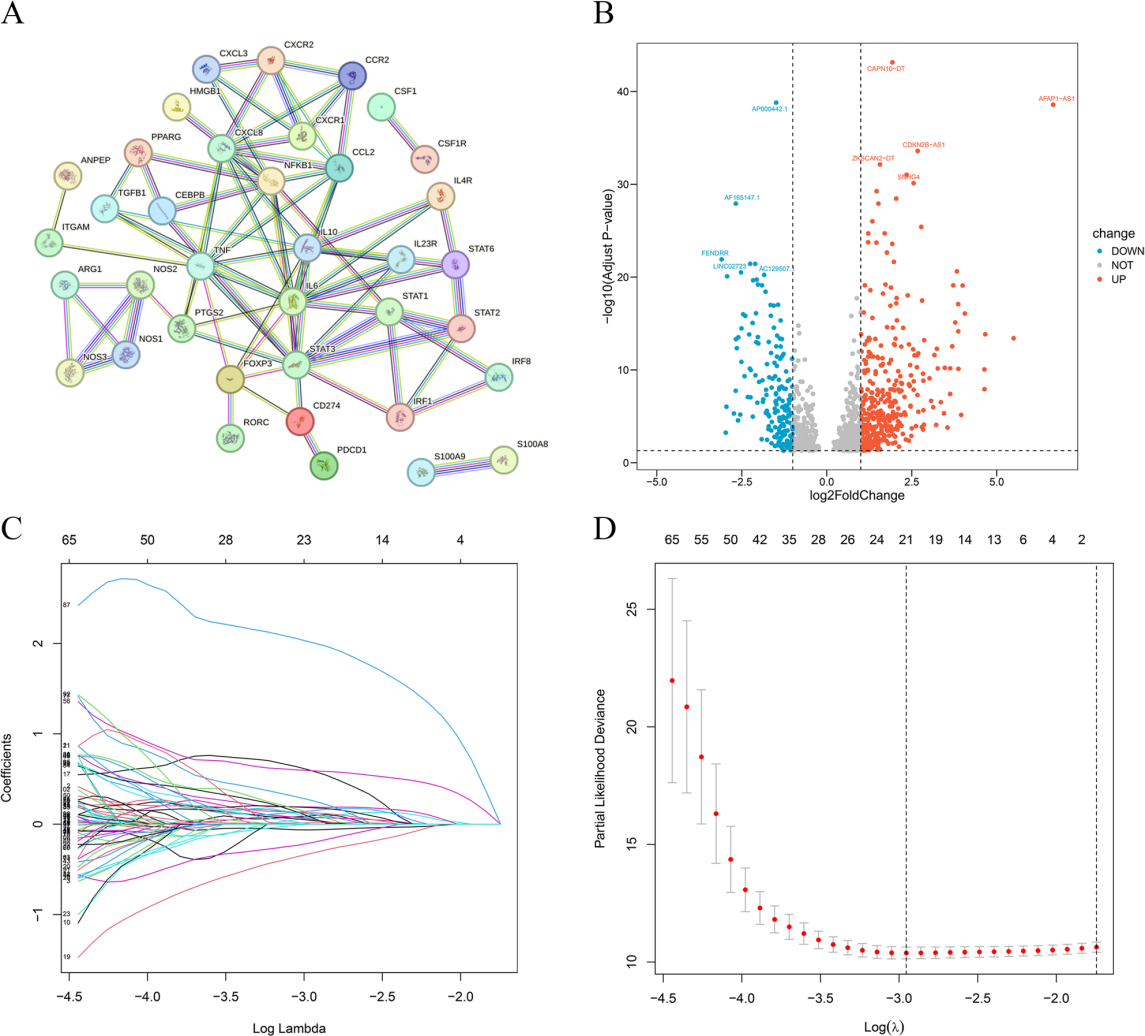
Filter for MDSCs-related lncRNAs. PPI network among 46 MDSCs-related genes (**A**); Volcano plot of differentially expressed MDSCs-associated lncRNAs (**B**); LASSO regression analysis (**C**-**D**)

Our research structure was depicted using a flowchart (Fig. 1). Firstly, MDSCs-related genes were retrieved, and a PPI network comprising 46 of all these genes was constructed by STRING database (Fig. 2A). Pearson correlation analysis was then performed, identifying 3,499 MDSCs-associated lncRNAs. This was further refined by differential expression analysis into 569 lncRNAs that exhibited significant differential expressions in tumor tissues (Fig. 2B).

Following differential analysis, randomly allocate all 369 HCC patients to training and testing formation (Table 1). Within the training team, the univariate Cox approach was applied to identify 120 lncRNAs notably tied to outcomes (*p* < 0.05) (Table S2). Subsequent LASSO regression further refined the selection to 21 differentially expressions of MDSCs-associated lncRNAs (Fig. 2C-D, Table S3). Multivariate Cox regression ultimately identified the 7 most significant lncRNAs (including AL365361.1, MSC-AS1, TMCC1-AS1, LINC02518, C3orf36, AC090510.2, and AC136297.1) (Figure S1A). Moreover, our team explored connections between 7 lncRNAs (Figure S1B) and their association with genes tied to MDSCs (Figure S1C).

**Table 1:** Clinical information of the patients in the test and training groups

Characteristics	Train cohort (*n* = 185)		Test cohort (*n* = 184)		Entire cohort (*n* = 369)	
	*n*	%	*n*	%	*n*	%
Age						
<=65	118	63.78	114	61.96	232	62.87
>65	67	36.22	70	38.04	137	37.13
Status						
Alive	117	63.24	122	66.3	239	64.77
Dead	68	36.76	62	33.7	150	35.23
Gender						
Female	64	34.59	57	30.98	121	32.79
Male	121	65.41	127	69.02	248	67.21
Stage						
Stage I	84	45.41	86	46.74	170	46.07
Stage II	44	23.78	41	22.28	85	23.04
Stage III	41	22.16	44	23.91	85	23.04
Stage IV	3	1.62	2	1.09	5	1.36
Unknow	13	7.03	11	5.98	24	6.5
T stage						
T1	90	48.65	90	48.91	180	48.78
T2	46	24.86	47	25.54	93	25.2
T3	39	21.08	41	22.28	80	21.68
T4	8	4.32	5	2.72	13	3.52
Unknow	2	0.54	1	0.54	3	0.81
M stage						
M0	135	72.97	130	70.65	265	71.82
M1	3	1.62	1	0.54	4	1.08
Unknow	47	25.41	53	28.8	100	27.1
N stage						
N0	126	68.11	125	67.93	251	68.02
N1	2	1.08	2	1.09	4	1.08
Unknow	57	30.81	57	30.98	114	30.89
Grade						
G1	33	17.84	22	11.96	55	14.91
G2	93	50.27	84	45.65	177	47.49
G3	50	27.03	70	38.04	120	32.52
G4	7	3.78	5	2.72	12	3.25
Unknow	2	1.08	3	1.63	5	1.36

*Abbreviations*: *T stage* Tumor stage, *N stage* Node stage, *M stage* Metastasis stage

This revealed the distinct patterns of upregulation and downregulation in tumor (Figure S1D).

### Establish and validating the prognostic model

**Fig. 3:**
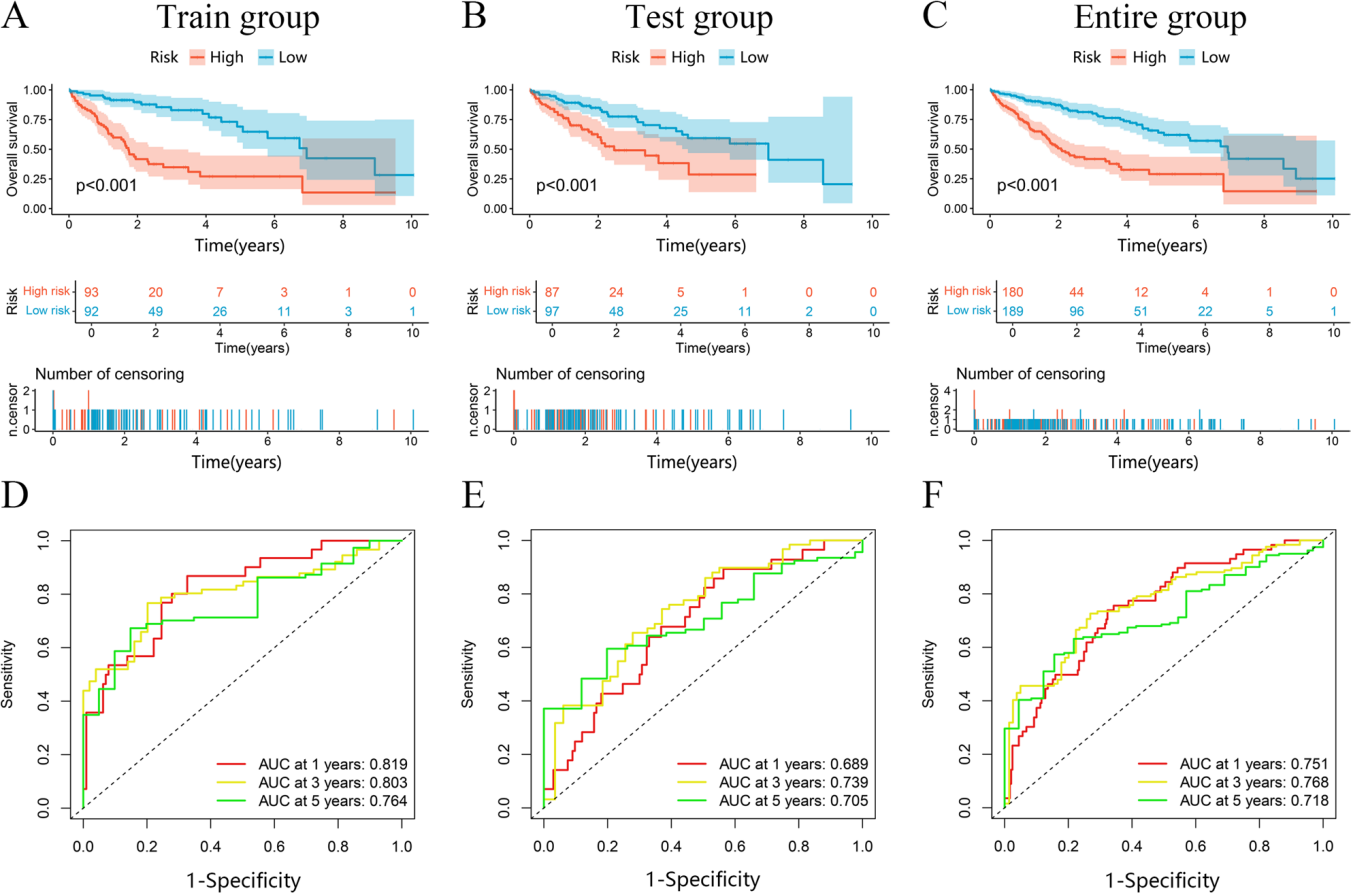
The model prediction effect is validated by the training group, test group, and entire group. K-M analysis (**A**-**C**) and Time-dependent ROC curves (**D**-**F**) to compare the survival of the high-risk group and low-risk group

Based on the 7 MDSCs-related lncRNAs, we calculated risk scores for different patients: risk score = expression(AL365361.1) * coefficient(-0.777762054333862)+expression(MSC-AS1) * coefficient(0.431746456278497)+expression(TMCC1-AS1) * coefficient(0.962019645890725)+expression(LINC02518) * coefficient(1.17284520101515)+expression(C3orf36) * coefficient(-0.742638520109038)+expression(AC090510.2) * coefficient(0.860057170454402)+expression(AC136297.1) * coefficient(2.49484643283722). The median score of two risk groups = 0.657. Table 2 presents the risk group distribution among patients with different clinical characteristics. Patients were grouped according to risk level, with survival disparities assessed across training, test, and full populations. K-M assessment showed extended survival among low-risk individuals (both *p* < 0.001) (Fig. 3A-C). Additionally, ROC plots indicated the model’s strong prediction capability, with AUC scores at 0.819, 0.799, and 0.761 for one-, three-, and five-year survival in the training group; 0.689, 0.739, and 0.705 in the test group; and 0.751, 0.768, and 0.718 in the merged group (Fig. 3D-F). The predictive effect of our model was further authenticated through risk score distribution curves, scatter plots, and risk heatmaps in different patient cohorts (Figure S2A-L).

**Table 2:** Clinical information for 369 patients in different risk categories

Characteristics	High-risk group (*n* = 180)		Low-risk group (*n* = 189)	
	*n*	%	*n*	%
Age				
<=65	115	63.89	117	61.9
>65	65	36.11	72	38.1
Status				
Alive	83	46.11	142	75.13
Dead	97	53.89	47	24.87
Gender				
Female	62	34.44	59	31.22
Male	118	65.56	130	68.78
Stage				
Stage I	67	37.22	103	54.5
Stage II	48	26.67	37	19.58
Stage III	55	30.56	30	15.87
Stage IV	2	1.11	3	1.59
Unknow	8	4.44	16	8.47
T stage				
T1	69	38.33	111	58.73
T2	54	30	39	20.63
T3	50	27.78	30	15.87
T4	7	3.89	6	3.17
Uknow	0	0	3	1.59
M stage				
M0	131	72.78	134	70.9
M1	2	1.11	2	1.06
Unknow	47	26.11	53	28.04
N stage				
N0	119	66.11	132	69.84
N1	3	1.67	1	0.53
Unknow	58	32.22	56	29.63
Grade				
G1	13	7.22	42	22.22
G2	79	43.89	98	51.85
G3	77	42.78	43	22.75
G4	10	5.56	2	1.06
Unknow	1	0.56	4	2.12

*Abbreviations*: *T stage* Tumor stage, *N stage* Node stage, *M stage* Metastasis stage

To provide external support at the expression level, we further examined the expression patterns of the final model-derived lncRNAs in GSE138178, GSE148355, and GSE214846. Consistent with the TCGA results, these lncRNAs showed reproducible differential expression trends between tumor and non-tumor/normal liver samples across the GEO cohorts (Figure S3). These findings support the cross-dataset stability of the expression patterns of the lncRNAs included in our signature.

**Fig. 4:**
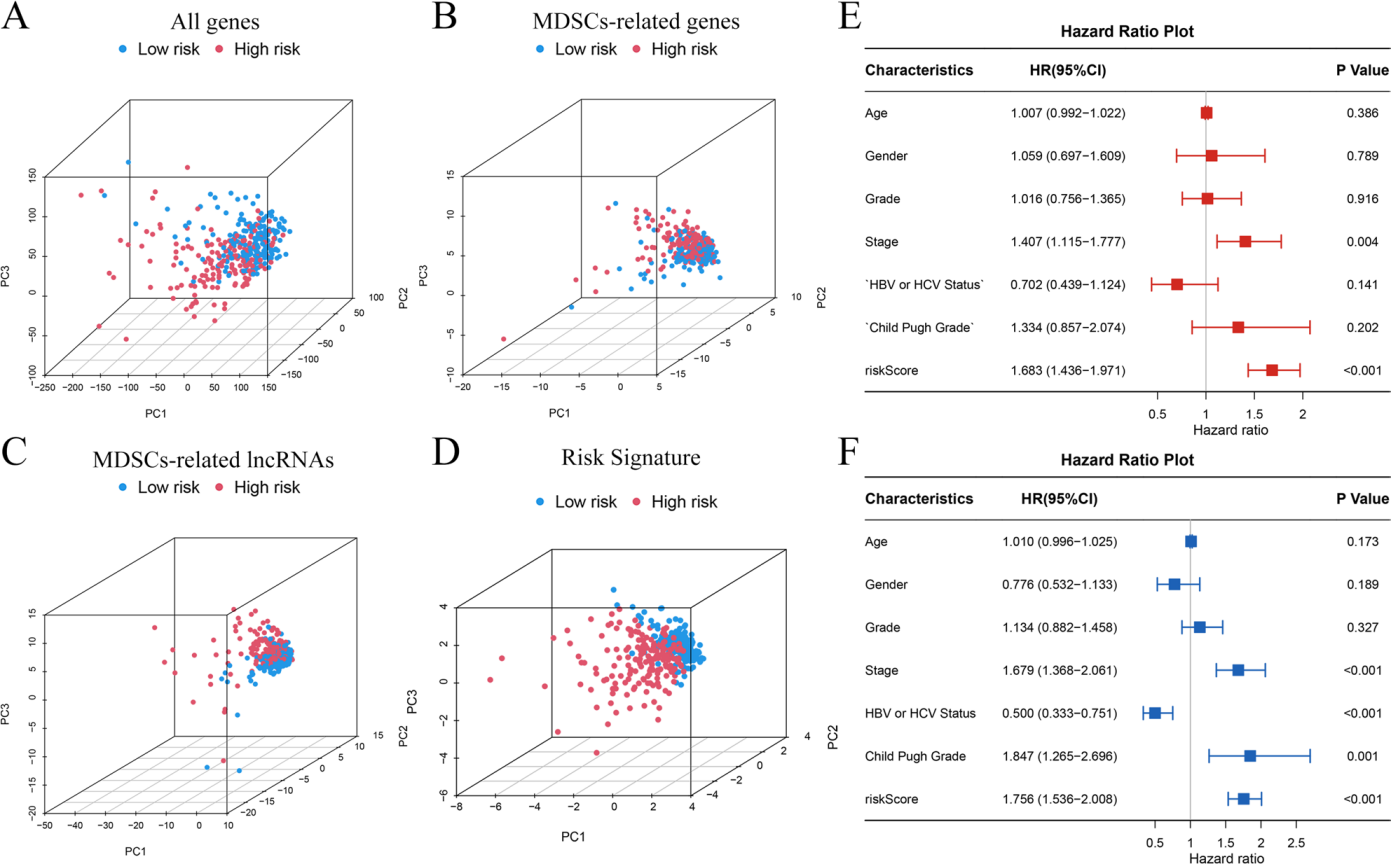
PCA and independent prognostic analysis of the signature. PCA based on all genes (**A**); all lncRNAs (**B**); MDSCs-related gene (**C**); and risk signature (**D**); Multivariate (**E**) and univariate (**F**) independent prognostic analysis

PCA (Fig. 4A-D) confirmed that patients between two different groups exhibited distinct clustering patterns. In multivariate Cox regression analysis, tumor stage (HR = 1.407, 95% CI = 1.115–1.777, *p* = 0.004) and risk score (HR = 1.683, 95% CI = 1.436–1.971, *p* < 0.001) were identified as independent prognostic factors (Fig. 4E). Univariate Cox regression analysis confirmed tumor stage (HR = 1.679, 95% CI = 1.368–2.061, *p* < 0.001), HBV or HCV Status (HR = 0.500, 95% CI = 0.333–0.751, *p* < 0.001), Child Pugh Grade (HR = 1.847, 95% CI = 1.265–2.696, *p* = 0.001) and risk score (HR = 1.756 95% CI = 1.536–2.008, *p* < 0.001) as independent predictors of overall survival (Fig. 4F).

**Fig. 5:**
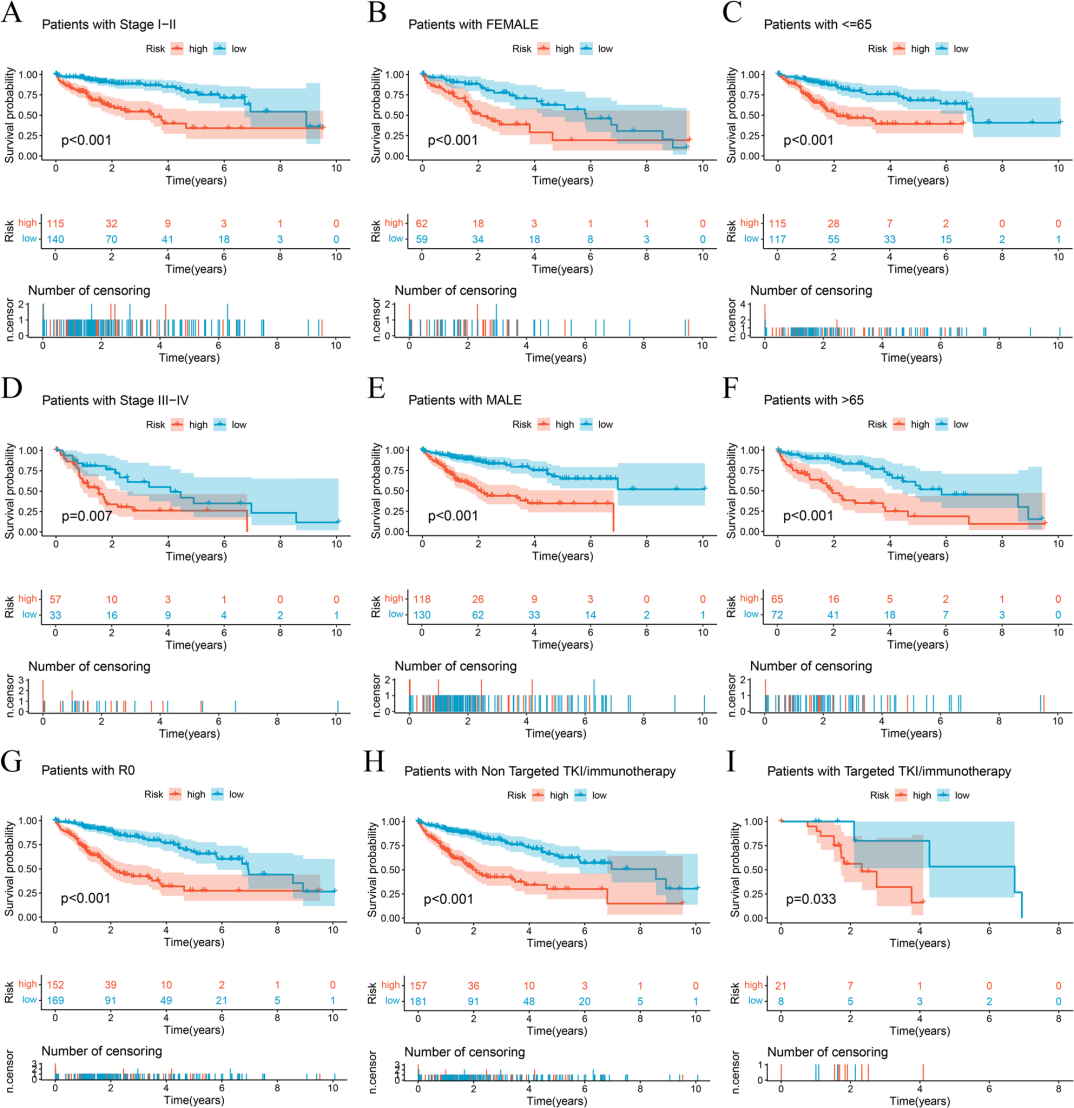
Further validation of model effects. Survival curves of patients in different clinical states (A-I).

We assessed the model’s prognostic reliability across diverse clinicopathologic subgroups to examine its consistency in varying clinical contexts. Heatmaps illustrating lncRNA expression levels, risk stratification, and clinical variables (Figure S4A) supported the model’s applicability. Survival analyses across different clinical subgroups further reinforced its predictive reliability (all *p* < 0.05) (Fig. 5).

### Predictive nomogram for clinical OS assessment

**Fig. 6:**
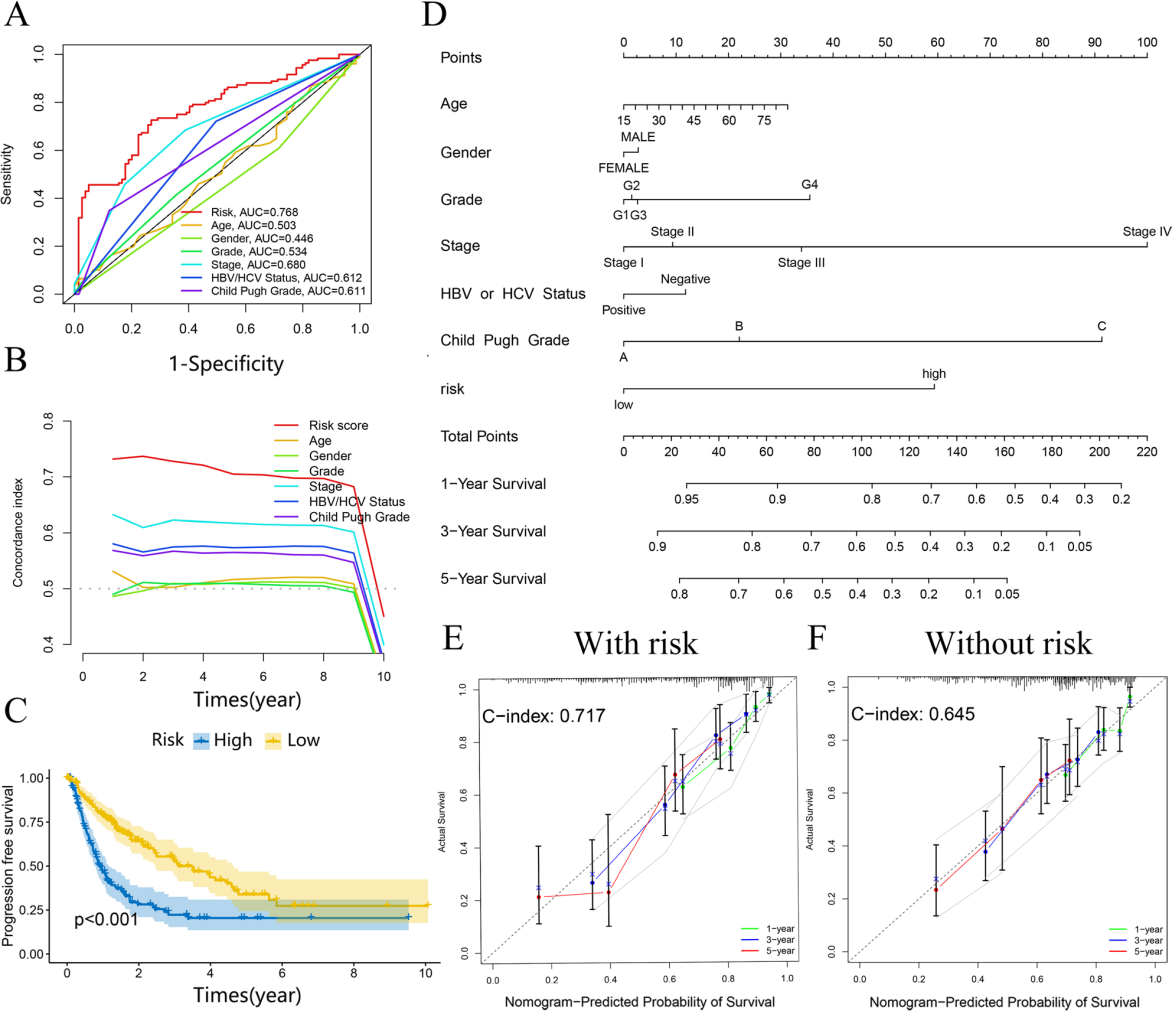
Nomogram predicts patient prognosis. ROC curves containing different clinical information (**A**); C-Index curve for the risk score and other clinical characteristics (**B**); PFS analysis (**C**); A clinical prognosis nomogram is constructed by age, HBV or HCV Status, child pugh grade, risk, grade, gender and stage together (**D**). Nomogram with (**E**) and without (**F**) risk model.

Findings confirmed a robust positive link involving risk score, tumor grade, and stage (*p* < 0.001), whereas its association with age and gender did not reach statistical significance (*p* > 0.05) (Figure S4B-E). ROC curve assessment validated the superior predictive ability of our prognostic model over most clinical parameters. The AUC of 0.768 for risk score, compared to 0.505 for age, 0.446 for sex, 0.534 for grade, and 0.680 for tumor stage (Fig. 6A). Additionally, the C-index curve (Fig. 6B) and PFS analysis (Fig. 6C) further substantiated the model’s superior prognostic value.

We developed a nomogram integrating risk value and tumor phase to estimate patient lifespan (Fig. 6D). Its predictive efficacy was validated using patient data, demonstrating strong performance (Figure S5A). Decision curve analysis (DCA) further evaluated the favorable net clinical benefit of the clinical factors and nomogram, underscoring its value in guiding individualized prognosis prediction (Figure S5B). Furthermore, comparison analysis revealed that the nomogram incorporating the risk model (Fig. 6E) had greater predictive accuracy than the one excluding the risk score (Fig. 6F).

### Enrichment analysis to elicit feature insight

KEGG enrichment analysis revealed critical roles of differentially expressed genes within cell cycle and bile production. And various metabolic pathways like ECM-receptor interaction or cytochrome P450 metabolism (Figure S6). GSEA further revealed distinct pathway enrichment patterns between different gene sets. Immune and metabolism-associated pathways were mainly concentrated in low-risk sets. High-risk individuals displayed elevated pathway activity tied to cell growth and DNA control. In the GOBP collection, pathways tied to amino acid processing, B-cell receptor activity, complement system stimulation, and monocarboxylic acid breakdown showed notable enrichment in low-risk patients (Figure S7). An extensive outline of pathway results emerges in Table S4.

### Tumor mutation burden

**Fig. 7:**
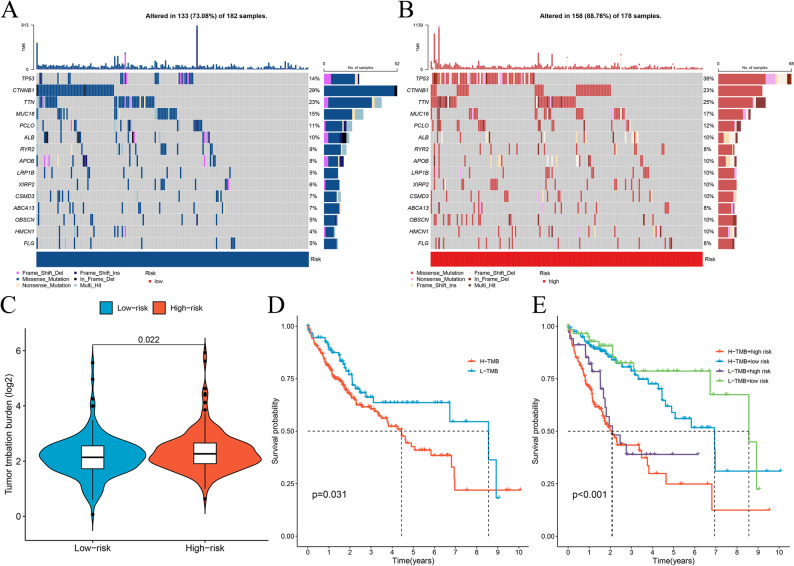
Tumor mutation burden in different risk groups. Low-risk group waterfall chart (**A**); High-risk group waterfall chart (**B**); TMB status in high risk and low risk group (**C**); The K–M curves show survival status and survival time in high-TMB and low-TMB (**D**); Stratified survival analysis (**E**)

TMB levels were assessed across patients, showing an increasing trend within the high-risk category (Fig. 7C). We further generated a waterfall plot to visualize the gene which were the 20 most frequently mutated in different subclusters (Fig. 7A, B). In populations at elevated risk, genes showing the highest mutation rates included TP53 (38%), TTN (25%), CTNNB1 (24%), MUC16 (17%) and PCLO (12%). Among individuals with lower risk, frequent genetic alterations appeared in CTNNB1 (29%), TTN (23%), and MUC16 (15%), followed by TP53 (14%) with PCLO (11%). Individuals underwent further grouping into elevated or reduced TMB clusters using median TMB figures. Survival outcomes revealed that individuals with reduced TMB had better OS (Fig. 7D). Analysis with TMB and risk scores revealed low-TMB survival benefits (Fig. 7E), aligning with our previous findings.

### Analysis of tumor immune penetration patterns

**Fig. 8:**
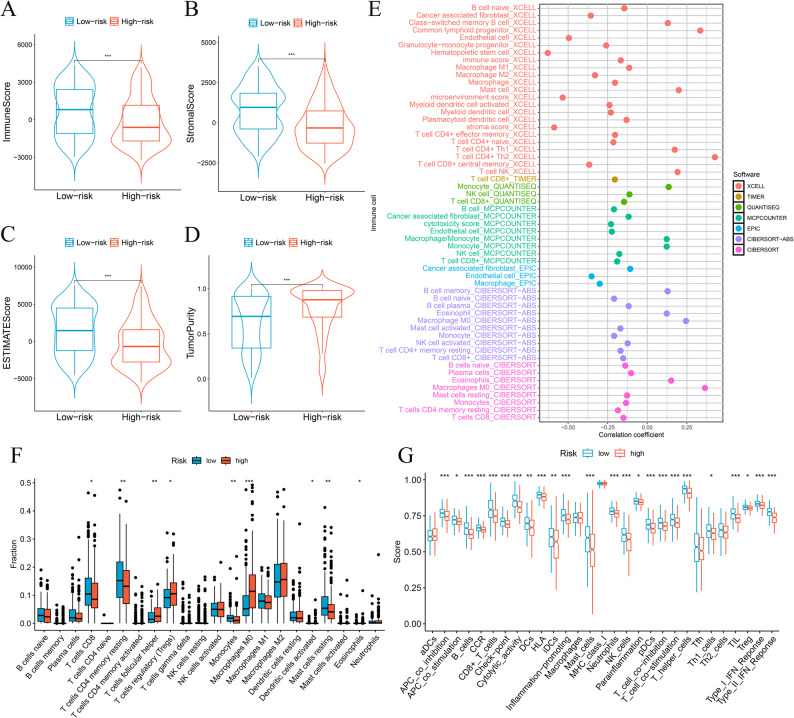
Analysis of tumor immune microenvironment. Violin plots of differences in immune scores (**A**), stromal scores (**B**), ESTIMATE scores (**C**), and tumor purity (**D**) for different risk subgroups; Bubble plots of correlations between immune cells and risk scores under six algorithms (**E**); Proportions of 22 immune cells in two subgroups under the CIBERSORT algorithm (**F**); single sample gene set enrichment analysis (**G**). *p < 0.05, **p < 0.01, ***p < 0.001

TME analysis indicated that patients with lower risk displayed notably higher immune (Fig. 8A), stromal (Fig. 8B), and ESTIMATE values (Fig. 8C) (*p* < 0.001). Conversely, the high-risk cohort showed substantially greater tumor purity (*p* < 0.001) (Fig. 8D). Results hint at better survival with low-risk immunity. The high-risk individual’s TME, characterized by immunosuppressive factors like MDSCs and immune evasion mechanisms, may lead to reduced immune infiltration. Moreover, diverse analytical methods assessed how immune infiltration correlates with risk levels (Fig. 8E). In the high-risk group, a significantly higher proportion of Tregs (*p* < 0.05), Macrophages M0 (*p* < 0.001), T follicular helper cells (*p* < 0.01), activated dendritic cells (*p* < 0.05), and eosinophils (*p* < 0.05) were observed. Conversely, the low-risk cohort displayed elevated levels of CD8 + T cells (*p* < 0.05), CD4 memory resting T cells (*p* < 0.01), resting mast cells (*p* < 0.01), and monocytes (*p* < 0.01) (Fig. 8F). Figure S8 illustrates immune cell abundance correlates with risk indicators. What’s more, immune function analysis indicated that all high-risk HCC patients exhibited immune suppression (Fig. 8G), which may contribute to tumor progression in this group.

We next assessed the relationship between the seven MDSC-associated lncRNAs and the MDSC score. Figure S9 show that TMCC1-AS1, AC090510.2, LINC02518, and AC136297.1 were positively correlated with the MDSC score (all *p* < 0.01), whereas AL365361.1 and C3orf36 showed significant negative correlations (both *p* < 0.001). MSC-AS1 displayed only a weak, non-significant association with the MDSC score (*p* = 0.074). Overall, these results support that most signature lncRNAs are closely linked to MDSC-related immune infiltration patterns in the HCC tumor microenvironment.

### Implications of the prognostic model in HCC treatment

TIDE analysis indicated that TIDE and MDSCs levels were notably elevated in high-risk group, along with a poor percentage of treatment responders. Conversely, Immunophenoscore (IPS) evaluation showed that CTLA-4 and PD-1 amounts were higher among reduced-risk subjects (Figure S10), implying decreased immune activity within tumors and weaker immunotherapy outcomes for higher-risk individuals.

Table S5 presents a selection of antitumor drugs with diverse mechanisms of action that show higher efficacy in low-risk patients, whereas Table S6 lists those that demonstrate greater effectiveness in high-risk patients. Agents with comparable sensitivity across both groups are outlined in Table S7.

Remarkably, the high-risk cohort displayed reduced responsiveness to medications affecting ERK and MAPK signaling, as well as DNA and chromosome activities. However, they exhibited increased responsiveness to inhibitors of the RTK and EGFR signaling pathways, which may provide valuable guidance for personalized selection of drugs.

### Subcellular localization analysis

To address the potential mechanistic implications of the identified lncRNAs, we further analyzed the subcellular localization of the seven MDSC-associated lncRNAs using lncATLAS and RNALocate (Figure S11)**.** TMCC1-AS1, MSC-AS1, and C3orf36 showed a predominant cytoplasmic distribution in the available cell lines. AL365361.1 exhibited a more variable distribution pattern across cell lines, indicating that its regulatory roles may be context-dependent. For AC090510.2, AC136297.1, and LINC02518, localization records from RNALocate suggested associations with extracellular vesicles or multiple subcellular compartments. Overall, these findings provide preliminary mechanistic clues that the seven lncRNAs may participate in HCC progression through distinct compartment-specific modes of action.

### Lab-based testing confirms risk predictions

**Fig. 9:**
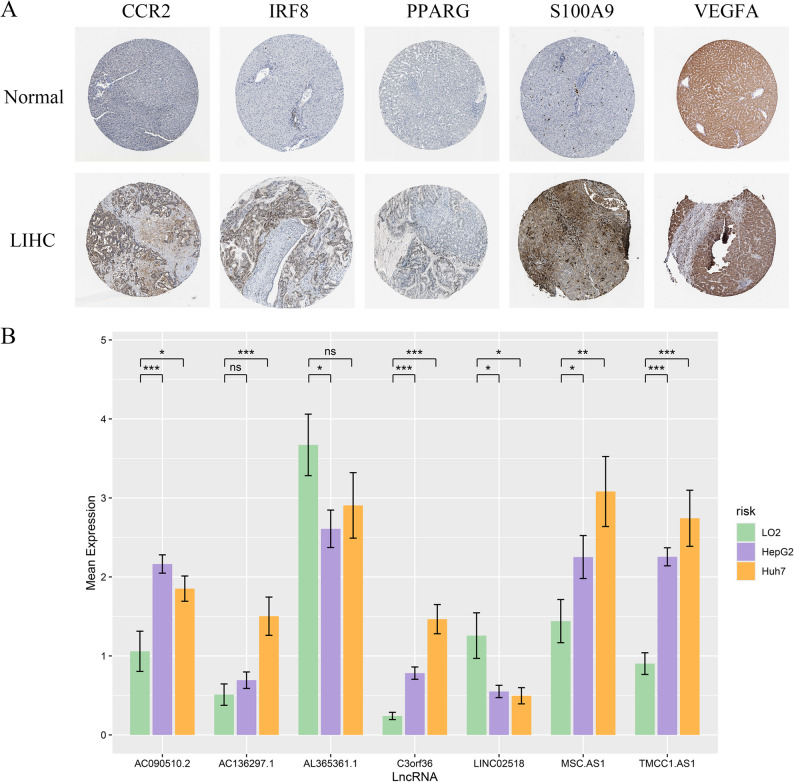
In vitro experimental validation of the risk model. Immunohistochemical staining images of partial MDSCs-associated gene proteins in HCC tissue and normal tissue (**A**); Relative expression of 7 MDSCs-related lncRNAs in different risk subgroups (**B**). *p < 0.05, **p < 0.01, ***p < 0.001, ****p < 0.0001

Staining visuals sourced from HPA showed MDSCs-related protein amounts in healthy and HCC samples (Fig. 9A). RT-qPCR data indicated where within the seven MDSCs-linked lncRNAs, AL365361.1 and LINC02518 were highly present in LO2 and additional five lncRNAs were lowly present compared to HepG2 and Huh7 cell lines. There was no significant difference between AC136297.1 in HepG2 and AL365361.1 in Huh7 (Fig. 9B).

## Discussion

HCC is a highly prevalent primary liver malignancy characterized by a high recurrence rate and poor prognosis [1]. Conventional TNM staging systems often fall short in predicting individualized outcomes, underscoring the need for integrative models that incorporate molecular markers [3]. Recent research highlights the critical roles of lncRNAs and MDSCs in tumor progression. Here, we constructed a prognostic model based on seven MDSC-associated lncRNAs, which accurately stratified HCC patients into distinct risk groups. Enrichment analyses indicated that metabolism- and immunity-related pathways were significantly enriched in the low-risk group, whereas high-risk patients were primarily associated with pathways involved in cell growth and genetic regulation. High-risk patients also exhibited elevated TMB, impaired immune function, and reduced predicted benefit from immunotherapy. Furthermore, drug-sensitivity analyses revealed distinct responses to targeted therapies between risk groups. Collectively, these findings suggest that our model not only provides a reliable prognostic tool for HCC but may also offer guidance for personalized treatment strategies.

A survival prediction tool built from seven MDSC-associated lncRNAs demonstrated strong prognostic performance, with high-risk patients showing markedly reduced OS time. Compared with the single-biomarker model reported by Bu et al. [23], our approach screened seven MDSC-related lncRNAs, thereby more comprehensively capturing molecular features associated with tumor survival and improving the model’s accuracy and biological relevance. Notably, time-dependent ROC analyses showed consistently high AUC values across time points (AUC = 0.768), outperforming the cuproptosis-related lncRNA model proposed by Peng et al. (AUC = 0.714), which highlights the superior performance of our signature for long-term survival prediction [24]. Moreover, our risk signature was validated across multiple clinical subgroups and exhibited stronger clinical generalizability than the ferroptosis-related model constructed by Xu et al. [25]. We further developed a nomogram integrating risk scores with multiple clinical variables to provide a more precise survival estimator (C-index: 0.716). Inclusion of the risk score significantly enhanced predictive accuracy and clinical utility compared with a nomogram without risk scores, and the model also outperformed the metabolic correlation model reported by Jia et al. (C-index: 0.667), further supporting its prognostic potential [26]. Among the lncRNAs identified, several have been reported in the literature for their involvement in cancer progression. In our study, MSC-AS1, TMCC1-AS1, and C3orf36 were predominantly localized in the cytoplasm, suggesting potential post-transcriptional regulatory functions such as ceRNA that may contribute to an immune-tolerant tumor microenvironment. MSC-AS1 has been reported to inhibit ovarian cancer progression via miR-425-5p [27], whereas it promotes hepatocarcinogenesis by inducing PGK1 expression in HCC, indicating context-dependent roles across tumor types and microenvironments [28]. TMCC1-AS1 has also been implicated in HCC malignancy, as its downregulation suppresses proliferation and migration [29]. Consistent with our finding that TMCC1-AS1 is positively correlated with the MDSC score, TMCC1-AS1–high tumors may be associated with a microenvironment favorable for MDSC accumulation and immune suppression. In addition, LINC02518 has been reported as an independent prognostic marker in HCC [30], and our results further support its potential association with an immunosuppressive niche. In contrast to strictly cytoplasmic lncRNAs, LINC02518, AC090510.2, and AC136297.1 showed multi-compartmental or extracellular vesicle–associated localization in our analysis, suggesting possible roles in intercellular communication within the tumor microenvironment. AC136297.1 has been linked to hypoxia-related pathways in glioblastoma and to elevated 5hmC-associated lncRNA signatures in pancreatic cancer [31, 32], implying potential relevance to stress-adaptive programs. Conversely, AL365361.1 and C3orf36 were negatively correlated with the MDSC score, suggesting potential protective roles. AL365361.1 has been reported to exert antitumor immune effects via the AL365361.1/miR-17-5p/NLRP3 axis [33], whereas C3orf36 has been observed to be downregulated in HCC [34] and associated with hypoxia-related risk in other tumors [35]. Although mechanistic evidence for AC090510.2 and AC136297.1 in HCC remains limited, their reported associations with ovarian and endometrial cancers warrant further functional validation in the context of MDSC-related regulation [36, 37]. Our research extends these investigations to hepatocellular carcinoma, revealing its broader implications in other malignancies. Using RT-qPCR, we validated the functional characteristics of seven long non-coding RNAs in malignant versus benign cells.

GSEA revealed that the high-risk cohort was dominated by cell proliferation and MYC activation, which drives both tumor growth and MDSC-mediated immune evasion [38]. In contrast, the low-risk group exhibited a robust immune-hot profile with higher CD8 + and CD4 + T cell infiltration, serving as the primary driver of superior clinical outcomes. The transition to a high-risk state likely represents a dynamic balance where immunosuppressive elements, such as Tregs and M0 macrophages [39–41], gradually outpace effector cells. Mechanistically, MDSCs in high-risk patients drive T-cell exclusion by depleting nutrients like arginine, producing reactive oxygen and nitrogen species, and indirectly augmenting Treg expansion through inhibitory cytokines like IL-10 and TGF-β [42, 43]. This suppressive niche is further reinforced by the upregulation of checkpoint molecules such as PD-1/PD-L1 or CTLA-4 and frequent TP53 mutations [44], which collectively facilitate the immune escape predicted by our TIDE analysis. This risk stratification provides a critical framework for the clinical management of immunotherapy’s effects and complications. High-risk individuals may require intensive monitoring of serum nutritional biomarkers [45] or closer surveillance for immune-related adverse events. Drawing from lessons in melanoma regarding cutaneous complications [46] and risks of immune-related kidney injury [47], our model identifies HCC patients who require proactive toxicity management or nutritional optimization to enhance the efficacy of checkpoint inhibitors. Furthermore, the inclusion of novel biomarkers like TMEM71 [48] reinforces the value of using MDSC-associated lncRNA signatures to provide a broader context for immune regulation and personalized treatment outcomes in liver cancer.

Our study also revealed substantial differences in predicted drug sensitivity between risk groups, providing a biologically plausible basis for precision treatment stratification in HCC. In the low-risk group, patients showed greater predicted sensitivity to MCL1 inhibitors such as AZD5991, suggesting that these tumors may be more vulnerable to apoptosis-based therapeutic strategies. This effect may arise not only from direct induction of mitochondrial apoptosis via inhibition of the anti-apoptotic protein MCL1, but also from potential effects of MCL1 blockade on the survival of immunosuppressive myeloid populations, including MDSCs, thereby reducing the suppressive capacity of the TME [49]. Sorafenib remains an important first-line targeted agent for HCC and primarily exerts antitumor activity through inhibition of the RAF–MEK–ERK pathway; however, prior studies suggest it may promote Treg-associated immunosuppressive changes that weaken therapeutic benefit [50]. In this context, our finding that low-risk patients exhibited a more favorable immune background and a higher predicted likelihood of benefiting from immune checkpoint blockade suggests that combining sorafenib with PD-1 inhibitors [51], including nivolumab or pembrolizumab, may represent a rational strategy to reinforce antitumor immunity by restoring effector T-cell activity while partially counteracting treatment-associated immune suppression [52]. By contrast, the high-risk group displayed a complex phenotype characterized by elevated TMB together with stronger immune exclusion and a more suppressive microenvironment, indicating that checkpoint blockade alone may be insufficient despite increased neoantigen load. Notably, these patients showed greater predicted sensitivity to savolitinib and BP-1-102, which target the HGF/MET and STAT3 pathways, respectively. Because these pathways are closely involved in MDSC recruitment, expansion, and functional maintenance, their inhibition may disrupt myeloid-driven immunosuppression, reduce immune exclusion, and improve immune-cell access to tumor tissue [53, 54]. Accordingly, combining PD-1 or CTLA-4 blockade with MET- or STAT3-targeted therapy may be particularly promising in high-risk patients by simultaneously suppressing oncogenic signaling, attenuating MDSC-mediated immune escape, and enhancing recognition of mutation-derived tumor antigens. Taken together, these findings suggest that the seven lncRNA risk signature may not only predict prognosis but also guide mechanism-based therapeutic selection by linking distinct immune and molecular states to differential drug susceptibility.

We validated our model by comparing it with similar studies, highlighting several strengths. First, this work originally explores links of MDSCs-associated lncRNAs to HCC patient prognosis, presenting these as pioneering indicators for outcome forecasting. Second, our risk signature and nomogram achieved higher AUC values and C-index than traditional models, demonstrating superior predictive accuracy. Moreover, the results of this model are consistent across different studies, indicating its robustness and reliability. Finally, it integrates immune function, IPS scores, and TIDE analyses to provide a more comprehensive insight of immune profile variations across risk cohorts. Still, this research has certain limitations. Firstly, this study is based on analyses of publicly accessible datasets for validation. Although we validated our signature using publicly available cohorts and observed consistent performance, several limitations remain. Second, as the analyses are largely retrospective and independent cohorts with complete seven-lncRNA profiles and well-annotated long-term outcomes are limited, prospective multicenter studies are needed to further confirm the model’s generalizability and its utility across different treatment strategies. What's more, the drug-sensitivity results were generated by in silico prediction frameworks and should be viewed as hypothesis-generating, requiring pharmacological validation in appropriate experimental models. Finally, while RT-qPCR verified the differential expression of the seven MDSC-associated lncRNAs, the absence of functional assays means their causal roles in MDSC regulation and HCC progression remain to be established.

## Conclusion

The model based on seven lncRNAs associated with MDSCs, demonstrating strong precision and practical use for predicting HCC survival. The high-risk cohort showed marked upregulation of DNA anomaly pathways, with reduced immune function and elevated TMB. In addition, we identified potential therapeutic agents to provide guidance for personalized treatment. However, additional testing and prospective clinical research are needed to verify the mechanistic insights and drug selection performance of the model.

## Supplementary Information

Supplementary Material 1.

Supplementary Material 2.

Supplementary Material 3.

Supplementary Material 4.

Supplementary Material 5.

Supplementary Material 6.

Supplementary Material 7.

Supplementary Material 8.

Supplementary Material 9.

Supplementary Material 10.

Supplementary Material 11.

Supplementary Material 12.

Supplementary Material 13.

Supplementary Material 14.

Supplementary Material 15.

Supplementary Material 16.

Supplementary Material 17.

Supplementary Material 18.

## Data Availability

Our data originates from publicly accessible databases, including the TCGA, TCIA, and HPA databases.

## Abbreviations

AUC: Area Under the Curve
CTLA-4: Cytotoxic T-Lymphocyte Associated Protein 4
DCA: Decision curve analysis
DMEM: Dulbecco’s Modified Eagle Medium
ESTIMATE: Estimation of Stromal and Immune cells in Malignant Tumor tissues using Expression data
GO: Gene Ontology
GOBP: Gene Ontology Biological Process
GSEA: Gene Set Enrichment Analysis
GSVA: Gene Set Variation Analysis
HCC: Hepatocellular Carcinoma
HPA: Human Protein Atlas
HR: Hazard Ratio
IC50: Half Maximal Inhibitory Concentration
IPS: Immunophenoscore
KEGG: Kyoto Encyclopedia of Genes and Genomes
K-M: Kaplan-Meier
LASSO: Least Absolute Shrinkage and Selection Operator
lncRNA: Long Noncoding RNA
MDSCs: Myeloid-derived Suppressor Cells
OS: Overall Survival
PCA: Principal Component Analysis
PD-1: Programmed Cell Death Protein 1
PFS: Progression-Free Survival
PPI: Protein-Protein Interaction
ROC: Receiver Operating Characteristic
RPMI-1640: Roswell Park Memorial Institute-1640
ssGSEA: Single-Sample Gene Set Enrichment Analysis
STRING: Search Tool for the Retrieval of Interacting Genes/Proteins
TCGA: The Cancer Genome Atlas
TIDE: Tumor Immune Dysfunction and Exclusion
TKIs: Tyrosine Kinase Inhibitors
TMB: Tumor Mutation Burden
TME: Tumor Microenvironment
Tregs: Regulatory T Cells
UCSC: University of California, Santa Cruz
