# Supplementary material for: Identification and external validation of a prognostic signature based on myeloid-derived suppressor cell-related lncRNAs for hepatocellular carcinoma
Source: Hereditas. 2026 Mar 19;163:54. doi: 10.1186/s41065-026-00664-z (PMC13123200; doi:10.1186/s41065-026-00664-z)
Supplement: Supplementary file 1 — Supplementary Material 1. [file 41065_2026_664_MOESM1_ESM.docx]

**Table S1** Primer sequences for 7 MDSCs-related lncRNAs.

| **Gene id** | **Primer F** | **Primer R** |
| --- | --- | --- |
| AL365361.1 | AACTCAGGTGACTCGCAGTG | AAAGATCCATGTGGGGCAGG |
| MSC-AS1 | GAACTGGGCTTGGACAGGATT | AGACAGCGAGACTGCGGC |
| TMCC1-AS1 | CGCTGCAGGTAGTGCTGAG | TGATATCCAAATTGGCAGTTGAT |
| LINC02518 | ACAAGGGGAGTCTCTGAGGA | GCATCAGGACAGCCAGTACA |
| C3orf36 | AACGCTCTTCACTCTGCTCC | TAAGTGTCCCGGTGAGCAAC |
| AC090510.2 | GAAGGCCTTTGGGACTTGGT | CGTGGTCAGGACAGGTTCC |
| AC136297.1 | ATCTGATTCTCGCCCTGTGG | GACGTGGCAACGTTAGAGGA |
