# Supplementary material for: Identification and external validation of a prognostic signature based on myeloid-derived suppressor cell-related lncRNAs for hepatocellular carcinoma
Source: Hereditas. 2026 Mar 19;163:54. doi: 10.1186/s41065-026-00664-z (PMC13123200; doi:10.1186/s41065-026-00664-z)
Supplement: Supplementary file 2 — Supplementary Material 2. [file 41065_2026_664_MOESM2_ESM.docx]

**Table S2** 120 differentially expressed MDSCs-related lncRNAs.

| **LncRNA** | **HR (95%Cl)** | **P-value** |  | **LncRNA** | **HR (95%Cl)** | **P-value** |
| --- | --- | --- | --- | --- | --- | --- |
| AC006369.1 | 0.34 (0.17-0.71) | 0.004 |  | Z97832.2 | 1.99 (1.07-3.69) | 0.029 |
| LINC01901 | 1.87 (1.01-3.45) | 0.045 |  | GRASLND | 2.14 (1.35-3.40) | 0.001 |
| CAPN10-DT | 1.70 (1.11-2.62) | 0.016 |  | AC011462.4 | 1.48 (1.08-2.02) | 0.016 |
| AC073842.2 | 1.91 (1.30-2.82) | 0.001 |  | MSC-AS1 | 1.48 (1.11-1.98) | 0.007 |
| AC006252.1 | 2.70 (1.66-4.41) | <0.001 |  | AC090241.3 | 6.53 (1.15-37.14) | 0.034 |
| AL606489.1 | 1.39 (1.08-1.79) | 0.010 |  | LINC00460 | 2.36 (1.05-5.29) | 0.037 |
| AC108463.2 | 2.00 (1.14-3.49) | 0.015 |  | AC107959.3 | 1.43 (1.11-1.83) | 0.005 |
| LINC00520 | 7.32 (1.34-39.89) | 0.021 |  | TFAP2A-AS1 | 1.45 (1.02-2.04) | 0.036 |
| SNHG1 | 1.36 (1.06-1.74) | 0.015 |  | PRR7-AS1 | 1.64 (1.06-2.53) | 0.026 |
| LHFPL3-AS1 | 2.44 (1.12-5.30) | 0.024 |  | TMCC1-AS1 | 2.94 (1.87-4.63) | <0.001 |
| AC068790.3 | 1.84 (1.03-3.29) | 0.039 |  | FOXCUT | 1.46 (1.04-2.05) | 0.027 |
| AL451069.3 | 1.39 (1.05-1.85) | 0.022 |  | AC093001.1 | 1.48 (1.01-2.17) | 0.043 |
| DHCR24-DT | 1.56 (1.08-2.24) | 0.018 |  | AC007255.1 | 2.53 (1.16-5.54) | 0.020 |
| AC004160.2 | 0.54 (0.31-0.93) | 0.026 |  | AC073611.1 | 7.85 (1.80-34.13) | 0.006 |
| AC090578.1 | 1.42 (1.11-1.82) | 0.006 |  | AL109615.2 | 4.22 (1.48-11.99) | 0.007 |
| AC025176.1 | 1.88 (1.33-2.66) | <0.001 |  | AC004837.4 | 3.35 (1.14-9.86) | 0.028 |
| AL445213.2 | 5.94 (1.61-21.91) | 0.007 |  | DDX11-AS1 | 3.18 (1.66-6.09) | <0.001 |
| AC022007.1 | 2.31 (1.53-3.48) | <0.001 |  | AC108463.3 | 2.32 (1.03-5.22) | 0.042 |
| AL365361.1 | 0.44 (0.24-0.80) | 0.007 |  | AC022092.1 | 1.97 (1.22-3.19) | 0.006 |
| AC096642.1 | 1.99 (1.05-3.77) | 0.035 |  | AL357079.1 | 2.66 (1.62-4.36) | <0.001 |
| WDFY3-AS2 | 2.05 (1.03-4.07) | 0.042 |  | AC023302.1 | 1.98 (1.14-3.43) | 0.015 |
| AC078909.2 | 1.44 (1.04-1.99) | 0.030 |  | LINC00894 | 1.95 (1.01-3.78) | 0.048 |
| AC097369.4 | 1.70 (1.05-2.77) | 0.033 |  | AC020915.1 | 1.40 (1.01-1.96) | 0.045 |
| LINC02820 | 1.34 (1.01-1.77) | 0.045 |  | AC108676.1 | 1.73 (1.01-2.96) | 0.047 |
| AC127024.5 | 1.67 (1.07-2.60) | 0.023 |  | ZNF775-AS1 | 1.79 (1.02-3.16) | 0.044 |
| AC026356.2 | 2.89 (1.17-7.14) | 0.021 |  | LENG8-AS1 | 1.52 (1.06-2.18) | 0.022 |
| LINC01579 | 4.28 (1.04-17.55) | 0.044 |  | AC092910.3 | 1.76 (1.07-2.91) | 0.027 |
| AC102953.2 | 1.48 (1.05-2.08) | 0.025 |  | AL513327.2 | 3.00 (1.43-6.31) | 0.004 |
| SNHG4 | 1.63 (1.28-2.07) | <0.001 |  | AC104024.4 | 4.70 (1.81-12.19) | 0.001 |
| TTC39A-AS1 | 2.53 (1.24-5.15) | 0.011 |  | CDKN2B-AS1 | 3.13 (1.21-8.08) | 0.019 |
| AC048344.4 | 3.26 (1.73-6.15) | <0.001 |  | LINC02518 | 3.56 (1.38-9.16) | 0.009 |
| LINC00205 | 1.59 (1.15-2.19) | 0.005 |  | LINC02345 | 3.15 (1.19-8.34) | 0.021 |
| AL132765.2 | 1.71 (1.02-2.87) | 0.042 |  | AC093928.1 | 2.36 (1.06-5.27) | 0.036 |
| AC010487.1 | 2.25 (1.45-3.50) | <0.001 |  | AL133410.1 | 2.48 (1.25-4.90) | 0.009 |
| AL590101.1 | 2.91 (1.28-6.60) | 0.011 |  | LINC01711 | 2.85 (1.25-6.50) | 0.013 |
| LINC02341 | 2.22 (1.31-3.74) | 0.003 |  | C3orf36 | 0.46 (0.23-0.91) | 0.027 |
| LINC02466 | 2.71 (1.01-7.29) | 0.049 |  | AC009121.2 | 3.49 (1.49-8.18) | 0.004 |
| AC010973.2 | 2.11 (1.18-3.79) | 0.012 |  | LINC00578 | 2.53 (1.20-5.31) | 0.014 |
| PVT1 | 1.74 (1.10-2.77) | 0.019 |  | FOXD2-AS1 | 1.48 (1.18-1.86) | 0.001 |
| AL355488.1 | 1.62 (1.23-2.12) | 0.001 |  | AC090510.2 | 2.18 (1.05-4.51) | 0.035 |
| AL117335.1 | 1.39 (1.05-1.86) | 0.023 |  | AC136297.1 | 18.08 (5.19-62.98) | <0.001 |
| LINC01594 | 2.11 (1.02-4.38) | 0.045 |  | AL138889.3 | 4.29 (1.27-14.48) | 0.019 |
| AP002852.1 | 2.88 (1.18-7.05) | 0.020 |  | LINC01649 | 1.77 (1.18-2.67) | 0.006 |
| AC024560.4 | 2.88 (1.51-5.49) | 0.001 |  | AC006144.2 | 1.65 (1.13-2.39) | 0.009 |
| AL353801.1 | 1.50 (1.02-2.22) | 0.042 |  | AC092667.1 | 1.73 (1.08-2.79) | 0.024 |
| AC012313.8 | 6.33 (1.60-25.05) | 0.009 |  | AC245060.6 | 2.68 (1.06-6.75) | 0.037 |
| AC106858.1 | 2.04 (1.10-3.78) | 0.023 |  | AL355388.1 | 1.98 (1.22-3.21) | 0.006 |
| LINC02870 | 1.61 (1.27-2.04) | <0.001 |  | AC083855.2 | 1.37 (1.05-1.78) | 0.020 |
| DDN-AS1 | 4.53 (1.43-14.38) | 0.010 |  | AC104335.2 | 1.51 (1.08-2.12) | 0.017 |
| AC084864.1 | 1.51 (1.02-2.24) | 0.041 |  | AC006557.1 | 3.02 (1.10-8.27) | 0.032 |
| LINC01436 | 1.15 (1.01-1.31) | 0.039 |  | AC104779.1 | 4.88 (1.62-14.70) | 0.005 |
| AC040970.1 | 1.30 (1.02-1.66) | 0.037 |  | LINC00622 | 1.41 (1.04-1.92) | 0.027 |
| AC005332.5 | 1.51 (1.10-2.07) | 0.010 |  | AL021392.1 | 3.01 (1.61-5.60) | 0.001 |
| AC025171.2 | 1.65 (1.08-2.53) | 0.021 |  | LHFPL3-AS2 | 1.27 (1.06-1.52) | 0.009 |
| AL359878.1 | 5.63 (2.34-13.54) | <0.001 |  | AC009902.3 | 10.60 (1.50-75.01) | 0.018 |
| AP000553.2 | 1.84 (1.18-2.86) | 0.007 |  | AL163952.1 | 2.06 (1.06-3.99) | 0.032 |
| LINC02709 | 1.67 (1.15-2.43) | 0.007 |  | AC093730.1 | 2.68 (1.28-5.61) | 0.009 |
| MIR4435-2HG | 2.18 (1.35-3.51) | 0.001 |  | LINC00862 | 1.99 (1.23-3.23) | 0.005 |
| AP003778.1 | 2.89 (1.51-5.51) | 0.001 |  | AC004816.1 | 1.44 (1.07-1.95) | 0.018 |
| AC011352.3 | 1.90 (1.18-3.06) | 0.009 |  | LINC00628 | 1.85 (1.04-3.29) | 0.035 |

**Abbreviations**: MDSCs: Myeloid-derived suppressor cells; HR: Hazard Ratio; Cl: Confidence level.
