# Supplementary material for: Identification and external validation of a prognostic signature based on myeloid-derived suppressor cell-related lncRNAs for hepatocellular carcinoma
Source: Hereditas. 2026 Mar 19;163:54. doi: 10.1186/s41065-026-00664-z (PMC13123200; doi:10.1186/s41065-026-00664-z)
Supplement: Supplementary file 3 — Supplementary Material 3. [file 41065_2026_664_MOESM3_ESM.docx]

**Table S3** 21 differentially expressed MDSCs-related lncRNAs.

| **LncRNA** | **Coef** |
| --- | --- |
| AC006369.1 | -0.0594139 |
| AC006252.1 | 0.0844847 |
| AC108463.2 | 0.0275224 |
| LINC00520 | 0.1076476 |
| AC090578.1 | 0.0705970 |
| AL445213.2 | 0.5442099 |
| AL365361.1 | -0.2857140 |
| AC048344.4 | 0.1634128 |
| AC010487.1 | 0.1857756 |
| LINC02341 | 0.1739327 |
| MSC-AS1 | 0.1179693 |
| TMCC1-AS1 | 0.5838541 |
| AC093001.1 | 0.0248384 |
| LINC02518 | 0.2192450 |
| LINC02345 | 0.0127985 |
| C3orf36 | -0.1713608 |
| LINC00578 | 0.0102730 |
| FOXD2-AS1 | 0.0169116 |
| AC090510.2 | 0.0688310 |
| AC136297.1 | 1.9718964 |
| AP003778.1 | 0.0934075 |

**Abbreviation:** MDSCs: Myeloid-derived suppressor cells.
