# Supplementary material for: Identification and external validation of a prognostic signature based on myeloid-derived suppressor cell-related lncRNAs for hepatocellular carcinoma
Source: Hereditas. 2026 Mar 19;163:54. doi: 10.1186/s41065-026-00664-z (PMC13123200; doi:10.1186/s41065-026-00664-z)
Supplement: Supplementary file 4 — Supplementary Material 4. [file 41065_2026_664_MOESM4_ESM.docx]

**Table S4** GSEA pathways for different risk groups.

| **Pathways** | **Group** |
| --- | --- |
| **C1.all.v2024.1.Hs.symbols.gmt** |  |
| chr19p12 | High risk |
| chr1q21 | High risk |
| chr2q35 | High risk |
| chrXp21 | High risk |
| chr7q32 | High risk |
| chrXq23 | High risk |
| chrXq28 | High risk |
| **C2.cp.wikipathways.v2024.1.Hs.symbols.gmt** |  |
| WP_ALLOGRAFT_REJECTION | Low risk |
| WP_COMPLEMENT_AND_COAGULATION_CASCADES | Low risk |
| WP_DENGUE2_INTERACTIONS_WITH_COMPLEMENT_AND_COAGULATION_CASCADES | Low risk |
| WP_EXTRAFOLLICULAR_AND_FOLLICULAR_B_CELL_ACTIVATION_BY_SARSCOV2 | Low risk |
| WP_OXYSTEROLS_DERIVED_FROM_CHOLESTEROL | Low risk |
| WP_CELL_CYCLE | High risk |
| WP_DNA_REPLICATION | High risk |
| WP_G1_TO_S_CELL_CYCLE_CONTROL | High risk |
| WP_PANCREATIC_CANCER_SUBTYPES | High risk |
| WP_RETINOBLASTOMA_GENE_IN_CANCER | High risk |
| **C4.cgn.v2024.1.Hs.symbols.gmt** |  |
| CAR_IGFBP1 | Low risk |
| GNF2_CEBPA | Low risk |
| GNF2_GSTM1 | Low risk |
| GNF2_HPN | Low risk |
| GNF2_HPX | Low risk |
| GNF2_LCAT | Low risk |
| GNF2_TST | Low risk |
| **C2.cp.pid.v2024.1.Hs.symbols.gmt** |  |
| PID_ATR_PATHWAY | High risk |
| PID_AURORA_B_PATHWAY | High risk |
| PID_E2F_PATHWAY | High risk |
| PID_FANCONI_PATHWAY | High risk |
| PID_FOXM1_PATHWAY | High risk |
| PID_MYC_ACTIV_PATHWAY | High risk |
| PID_PLK1_PATHWAY | High risk |
| **C5.go.bp.v2024.1.Hs.symbols.gmt** |  |
| GOBP_AMINO_ACID_CATABOLIC_PROCESS | Low risk |
| GOBP_B_CELL_RECEPTOR_SIGNALING_PATHWAY | Low risk |
| GOBP_COMPLEMENT_ACTIVATION | Low risk |
| GOBP_COMPLEMENT_ACTIVATION_CLASSICAL_PATHWAY | Low risk |
| GOBP_L_AMINO_ACID_CATABOLIC_PROCESS | Low risk |
| **C7.immunesigdb.v2024.1.Hs.symbols.gmt** |  |
| GSE13547_CTRL_VS_ANTI_IGM_STIM_BCELL_12H_UP | High risk |
| GSE14415_NATURAL_TREG_VS_TCONV_DN | High risk |
| GSE15750_DAY6_VS_DAY10_EFF_CD8_TCELL_UP | High risk |
| GSE15750_DAY6_VS_DAY10_TRAF6KO_EFF_CD8_TCELL_UP | High risk |
| GSE27241_WT_VS_RORGT_KO_TH17_POLARIZED_CD4_TCELL_UP | High risk |
| GSE30962_PRIMARY_VS_SECONDARY_ACUTE_LCMV_INF_CD8_TCELL_UP | High risk |
| GSE45365_WT_VS_IFNAR_KO_CD11B_DC_MCMV_INFECTION_DN | High risk |
| **C2.cp.biocarta.v2024.1.Hs.symbols.gmt** |  |
| BIOCARTA_AMI_PATHWAY | Low risk |
| BIOCARTA_COMP_PATHWAY | Low risk |
| BIOCARTA_CSK_PATHWAY | Low risk |
| BIOCARTA_CTLA4_PATHWAY | Low risk |
| BIOCARTA_INTRINSIC_PATHWAY | Low risk |
| BIOCARTA_NO2IL12_PATHWAY | Low risk |
| BIOCARTA_NUCLEARRS_PATHWAY | Low risk |

**Abbreviation:** GSEA: Gene Set Enrichment Analysis.
