# Supplementary material for: Identification and external validation of a prognostic signature based on myeloid-derived suppressor cell-related lncRNAs for hepatocellular carcinoma
Source: Hereditas. 2026 Mar 19;163:54. doi: 10.1186/s41065-026-00664-z (PMC13123200; doi:10.1186/s41065-026-00664-z)
Supplement: Supplementary file 5 — Supplementary Material 5. [file 41065_2026_664_MOESM5_ESM.docx]

**Table S5** Antineoplastic drug sensitivity information (sensitive group: low).

| **Target pathways** | **Low-risk group** |  | **High-risk group** | **P-value** |
| --- | --- | --- | --- | --- |
|  | **IC50 (25%-75%）** |  | **IC50 (25%-75%）** |  |
| **Apoptosis regulation** |  |  |  |  |
| AZD5991 | 45.4 (11.09-143.17) |  | 75.96 (15.86-327.29) | 0.004 |
| Obatoclax Mesylate | 2.34 (1.15-6.28) |  | 6 (2.48-16.08) | <0.0001 |
| Sabutoclax | 0.35 (0.15-0.96) |  | 0.78 (0.27-3.34) | <0.0001 |
| **Cell cycle** |  |  |  |  |
| CDK9_5038 | 0.03 (0.01-0.13) |  | 0.06 (0.01-0.62) | 0.04 |
| CDK9_5576 | 0.27 (0.12-0.83) |  | 0.46 (0.13-2.3) | 0.03 |
| Palbociclib | 33.38 (21.82-53.39) |  | 49.04 (27.76-82.44) | <0.0001 |
| Ribociclib | 42.03 (37.51-49.28) |  | 51.58 (42.85-62.98) | <0.0001 |
| **Chromatin histone acetylation** |  |  |  |  |
| Entinostat | 7.82 (4.12-16.37) |  | 10.91 (6.01-19) | 0.002 |
| OF-1 | 35.02 (19.31-69.44) |  | 74.49 (37.26-185.76) | <0.0001 |
| PCI-34051 | 41.9 (24.13-100.55) |  | 84.87 (37.23-333.87) | <0.0001 |
| GSK591 | 91.44 (64.47-129.15) |  | 113.15 (79.44-163.33) | 0.001 |
| **Chromatin other** |  |  |  |  |
| AZD5153 | 4.85 (1.59-10.87) |  | 7.57 (3.34-16.34) | <0.0001 |
| I-BET-762 | 26.77 (11.36-44.38) |  | 34.2 (19.21-69.05) | 0.0001 |
| JQ1 | 7.04 (3.19-13.26) |  | 17.65 (6.62-41.49) | <0.0001 |
| OTX015 | 12.63 (5.07-23.16) |  | 15.5 (8-26.59) | 0.01 |
| **Cytoskeleton** |  |  |  |  |
| PAK_5339 | 9.96 (6.64-14.14) |  | 12.45 (8.97-18.24) | <0.0001 |
| **DNA replication** |  |  |  |  |
| Camptothecin | 0.05 (0.02-0.15) |  | 0.17 (0.05-0.6) | <0.0001 |
| Cisplatin | 17.53 (6.43-42.88) |  | 33.85 (13.82-88.19) | <0.0001 |
| Epirubicin | 0.26 (0.1-0.72) |  | 0.45 (0.11-2.45) | 0.008 |
| Fludarabine | 58.03 (18.86-186.43) |  | 176 (47.28-619.57) | <0.0001 |
| Gemcitabine | 0.25 (0.11-0.78) |  | 1.03 (0.31-2.64) | <0.0001 |
| Irinotecan | 6.7 (2.4-19.43) |  | 25.94 (8.8-78.79) | <0.0001 |
| Leflunomide | 119.27 (87.4-176.53) |  | 155.88 (108.07-259.93) | <0.0001 |
| Mitoxantrone | 0.66 (0.24-2.62) |  | 2.81 (0.65-12.43) | <0.0001 |
| Nelarabine | 216.21 (97.68-476.44) |  | 411.46 (176.77-1424.88) | <0.0001 |
| Oxaliplatin | 31.81 (18.2-53.41) |  | 55.68 (27.95-104.34) | <0.0001 |
| Teniposide | 0.54 (0.13-3.68) |  | 2.73 (0.51-23.99) | <0.0001 |
| Topotecan | 0.43 (0.1-1.96) |  | 2.55 (0.58-11.54) | <0.0001 |
| **EGFR signaling** |  |  |  |  |
| AZD3759 | 13.29 (8.2-21.2) |  | 16.58 (8.41-28.63) | 0.02 |
| **ERK MAPK signaling** |  |  |  |  |
| Dabrafenib | 91.78 (46-155.27) |  | 112.03 (60.38-219.07) | 0.006 |
| ERK_2440 | 5.34 (1.4-21.21) |  | 16.16 (4.14-123.27) | <0.0001 |
| ERK_6604 | 27.07 (6.11-70.48) |  | 34.22 (13.25-115.62) | 0.001 |
| KRAS (G12C) Inhibitor-12 | 18.72 (5.27-126.23) |  | 144.42 (23.37-1435.13) | <0.0001 |
| PD0325901 | 1.44 (0.63-2.97) |  | 1.97 (1.07-3.66) | 0.003 |
| PLX-4720 | 45.04 (22.19-101.32) |  | 142.72 (55.85-288.15) | <0.0001 |
| Selumetinib | 38.75 (10.78-113.85) |  | 102.3 (37.86-301.31) | <0.0001 |
| **Genome integrity** |  |  |  |  |
| Mirin | 67.31 (43.53-136.26) |  | 131.74 (66.94-392.29) | <0.0001 |
| Niraparib | 53.3 (31.11-92.55) |  | 91.48 (43.71-172.15) | <0.0001 |
| NU7441 | 12.8 (7.55-18.73) |  | 16.04 (10.64-23.29) | 0.0001 |
| Olaparib | 55.41 (33.89-93.5) |  | 84.8 (43.08-163.85) | 0.0003 |
| Talazoparib | 25.03 (10.37-43.93) |  | 32.26 (14.71-55.95) | 0.02 |
| **Hormone-related** |  |  |  |  |
| Tamoxifen | 33.3 (23.92-45.06) |  | 36.05 (24.79-60.11) | 0.02 |
| **IGF1R signaling** |  |  |  |  |
| BMS-754807 | 1.18 (0.56-2.05) |  | 1.69 (0.91-3.87) | <0.0001 |
| GSK1904529A | 51.68 (31.99-100.04) |  | 74.4 (34.52-225.35) | 0.001 |
| IGF1R_3801 | 4.41 (1.32-9.85) |  | 10.06 (3.92-23.07) | <0.0001 |
| Linsitinib | 40.94 (24.16-66.87) |  | 47.08 (27.96-74.72) | 0.04 |
| **JNK and p38 signaling** |  |  |  |  |
| Doramapimod | 79.94 (54.1-121.82) |  | 105.16 (75.42-156.11) | <0.0001 |
| **Metabolism** |  |  |  |  |
| AGI-6780 | 43.42 (25.36-89.91) |  | 64.81 (31.83-159.2) | 0.001 |
| GSK2606414 | 35.51 (17.44-60.48) |  | 57.84 (32.28-102.91) | <0.0001 |
| **Mitosis** |  |  |  |  |
| Alisertib | 5.76 (1.28-21.59) |  | 8.83 (2.55-31.31) | 0.03 |
| ZM447439 | 15.34 (9.89-24.12) |  | 17.96 (12.1-40.24) | 0.003 |
| **Other** |  |  |  |  |
| BMS-345541 | 14.01 (7.08-40.78) |  | 36.64 (13.04-148.48) | <0.0001 |
| Cytarabine | 3.32 (1.27-8.33) |  | 8.1 (3.48-19.26) | <0.0001 |
| Eg5_9814 | 0.02 (0.01-0.08) |  | 0.04 (0.01-0.34) | 0.01 |
| LY2109761 | 107.34 (51-286.67) |  | 173.36 (61.84-516.59) | 0.009 |
| Picolinici-acid | 143.31 (104.77-217.32) |  | 171.54 (118.19-271.08) | 0.004 |
| TAF1_5496 | 18.82 (6.2-63.25) |  | 74.37 (20.4-289.05) | <0.0001 |
| **Other, kinases** |  |  |  |  |
| AZ960 | 4.96 (2.44-12.32) |  | 9.19 (4.52-20.22) | <0.0001 |
| AZD1208 | 184.12 (124.81-251.37) |  | 208.92 (152.91-303.18) | 0.003 |
| AZD5363 | 17.97 (8.19-32.95) |  | 22.11 (11.49-39.2) | 0.04 |
| Entospletinib | 31.51 (20.35-50.26) |  | 52.56 (28.24-89.14) | <0.0001 |
| GSK2578215A | 125 (92.22-165.18) |  | 142.53 (108.88-216.5) | 0.0001 |
| Ibrutinib | 80.15 (42.32-129.43) |  | 85.51 (46.31-203.47) | 0.03 |
| IRAK4_4710 | 101.43 (59.25-168.47) |  | 174.38 (100.79-290.31) | <0.0001 |
| JAK_8517 | 12.59 (3.51-39.86) |  | 32.6 (10.8-140.89) | <0.0001 |
| JAK1_8709 | 39.85 (14.27-76.78) |  | 105.9 (49.24-230.32) | <0.0001 |
| Ruxolitinib | 114.02 (78.44-160.24) |  | 132.95 (102.59-184.52) | 0.0002 |
| Sorafenib | 10.38 (7.52-16.29) |  | 15.38 (9.27-26.49) | <0.0001 |
| **p53 pathway** |  |  |  |  |
| Nutlin-3a (-) | 64.76 (12.29-126.55) |  | 204.75 (79.2-504.31) | <0.0001 |
| **PI3K/MTOR signaling** |  |  |  |  |
| AT13148 | 28.36 (12.68-60.2) |  | 50.87 (29.53-96.98) | <0.0001 |
| AZD2014 | 5.72 (2.54-12.27) |  | 13.19 (6.29-30.14) | <0.0001 |
| AZD8055 | 0.75 (0.55-0.96) |  | 0.88 (0.59-1.19) | 0.0003 |
| Dactolisib | 0.18 (0.13-0.25) |  | 0.24 (0.16-0.34) | <0.0001 |
| LJI308 | 153.67 (107.02-222.79) |  | 188.88 (121.8-277.02) | 0.003 |
| Rapamycin | 0.08 (0.04-0.19) |  | 0.14 (0.04-0.3) | 0.01 |
| **Protein stability and degradation** |  |  |  |  |
| Luminespib | 0.06 (0.02-0.16) |  | 0.16 (0.05-0.75) | <0.0001 |
| ML323 | 70.85 (45.36-122.78) |  | 95.13 (52.64-166.9) | 0.005 |
| **RTK signaling** |  |  |  |  |
| Axitinib | 16.58 (11.21-25.1) |  | 26.48 (17.55-43.97) | <0.0001 |
| AZD1332 | 32.85 (12.18-63.61) |  | 80.04 (35.75-194.22) | <0.0001 |
| **WNT signaling** |  |  |  |  |
| LGK974 | 37.57 (27.09-63.91) |  | 62.57 (32.33-143.02) | <0.0001 |
| SB216763 | 147.92 (121.14-208.71) |  | 191.67 (138.14-311.65) | <0.0001 |
| Wnt-C59 | 43.17 (23.77-91.31) |  | 114.55 (39.21-266.71) | <0.0001 |
| **Unclassified** |  |  |  |  |
| Dihydrorotenone | 1.22 (0.49-3.28) |  | 3.44 (1.35-9.31) | <0.0001 |
| Elephantin | 7.82 (3.08-43.1) |  | 20.15 (4.71-549.94) | 0.0004 |
| Podophyllotoxin bromide | 0.22 (0.1-0.7) |  | 0.41 (0.13-1.81) | 0.002 |
| Sinularin | 23.72 (12.96-57.21) |  | 35.87 (16.36-89.85) | 0.004 |
| Vincristine | 0.04 (0.01-0.58) |  | 0.11 (0.01-4.24) | 0.01 |
| GSK269962A | 14.01 (8.7-23.17) |  | 23.55 (15.65-36.98) | <0.0001 |
| PF-4708671 | 30.3 (17.41-55.16) |  | 65.32 (36.52-132.88) | <0.0001 |
| SB505124 | 7.36 (5.66-9.89) |  | 10.34 (7.48-14.95) | <0.0001 |

**Abbreviation:** IC50: half maximal inhibitory concentration.
