# Supplementary material for: Identification and external validation of a prognostic signature based on myeloid-derived suppressor cell-related lncRNAs for hepatocellular carcinoma
Source: Hereditas. 2026 Mar 19;163:54. doi: 10.1186/s41065-026-00664-z (PMC13123200; doi:10.1186/s41065-026-00664-z)
Supplement: Supplementary file 6 — Supplementary Material 6. [file 41065_2026_664_MOESM6_ESM.docx]

**Table S6** Antineoplastic drug sensitivity information (sensitive group: high).

| **Target pathways** | **Low-risk group** |  | **High-risk group** | **P-value** |
| --- | --- | --- | --- | --- |
|  | **IC50 (25%-75%）** |  | **IC50 (25%-75%）** |  |
| **ABL signaling** |  |  |  |  |
| Nilotinib | 44.58 (21.9-76.14) |  | 33 (17.78-57.97) | 0.02 |
| **Apoptosis regulation** |  |  |  |  |
| ABT737 | 12.34 (4.15-34.19) |  | 8.04 (1.53-22.18) | 0.001 |
| Navitoclax | 9.23 (3.08-24.03) |  | 5.39 (1.52-17.95) | 0.003 |
| Sepantronium bromide | 0.01 (0-0.07) |  | 0.01 (0-0.05) | 0.03 |
| **Cell cycle** |  |  |  |  |
| AZD7762 | 1.82 (0.69-5.96) |  | 0.87 (0.26-3.4) | 0.0001 |
| MK-8776 | 34.23 (16.9-78.99) |  | 16.98 (6.62-38) | <0.0001 |
| Wee1 Inhibitor | 10.73 (4.44-28.95) |  | 6.04 (2.02-19.71) | 0.005 |
| **Chromatin other** |  |  |  |  |
| I-BRD9 | 98.04 (67.11-145.67) |  | 80.97 (42.32-120.89) | 0.0009 |
| PFI3 | 202.49 (153.96-265.69) |  | 179.62 (131.25-243.11) | 0.02 |
| **Cytoskeleton** |  |  |  |  |
| BDP-00009066 | 14.9 (9.2-23.37) |  | 10.33 (5.22-19.97) | 0.0006 |
| **EGFR signaling** |  |  |  |  |
| Afatinib | 9.66 (4.37-16.05) |  | 6.24 (2.41-11.39) | 0.0003 |
| Gefitinib | 34.09 (14.98-86.53) |  | 19.64 (7.61-44.38) | <0.0001 |
| Lapatinib | 27.53 (15.04-47.97) |  | 17.16 (7.04-32.24) | <0.0001 |
| Osimertinib | 8.57 (3.69-14.84) |  | 5 (2-9.66) | <0.0001 |
| Sapitinib | 67.03 (38.48-100.75) |  | 52.51 (27.97-96.61) | 0.03 |
| **ERK MAPK signaling** |  |  |  |  |
| VX-11e | 22.33 (12.17-39.12) |  | 16.57 (5.62-33.77) | 0.001 |
| **Hormone-related** |  |  |  |  |
| Fulvestrant | 23.99 (15.76-38.24) |  | 14.86 (8.92-23.78) | <0.0001 |
| GDC0810 | 192.89 (125.08-252.32) |  | 114.67 (65.8-168.64) | <0.0001 |
| **IGF1R signaling** |  |  |  |  |
| BMS-536924 | 10.14 (5.23-20.85) |  | 7.85 (3.14-15.66) | 0.002 |
| **Metabolism** |  |  |  |  |
| Daporinad | 0.02 (0.01-0.05) |  | 0.01 (0-0.05) | 0.007 |
| **Mitosis** |  |  |  |  |
| Docetaxel | 0.02 (0.01-0.05) |  | 0.01 (0-0.03) | 0.0001 |
| Paclitaxel | 0.17 (0.03-0.73) |  | 0.04 (0.01-0.2) | <0.0001 |
| Vinorelbine | 0.07 (0.03-0.17) |  | 0.05 (0.01-0.13) | 0.03 |
| **Other** |  |  |  |  |
| 5-Fluorouracil | 158.31 (73.58-334.65) |  | 94.48 (36.6-258.92) | 0.0002 |
| BPD-00008900 | 110.34 (77.03-165.43) |  | 87.07 (52.04-138.46) | 0.0001 |
| Pevonedistat | 2.29 (1.28-4.24) |  | 1.8 (0.58-4.28) | 0.02 |
| YK-4-279 | 15.92 (7.38-29.52) |  | 9.74 (3.28-19.87) | <0.0001 |
| **Other, kinases** |  |  |  |  |
| Dasatinib | 14.65 (3.65-34.05) |  | 5.02 (0.3-18.86) | <0.0001 |
| **p53 pathway** |  |  |  |  |
| MIRA-1 | 288.83 (194.15-393.56) |  | 191.83 (123.77-314.28) | <0.0001 |
| **PI3K/MTOR signaling** |  |  |  |  |
| Alpelisib | 49.26 (25.81-87.19) |  | 32.52 (16.24-60.32) | <0.0001 |
| AMG-319 | 139.69 (100.49-208.49) |  | 121.48 (75.09-189.11) | 0.02 |
| GNE-317 | 1.96 (1.49-2.81) |  | 1.52 (0.98-2.59) | 0.0002 |
| Ipatasertib | 57.78 (23.85-106.15) |  | 25.5 (9.19-73.21) | <0.0001 |
| Pictilisib | 6.29 (2.9-11.12) |  | 3.78 (1.51-7.9) | <0.0001 |
| Taselisib | 16.23 (7.14-35.73) |  | 6.13 (1.77-17.07) | <0.0001 |
| **RTK signaling** |  |  |  |  |
| Cediranib | 9.95 (6.22-19.79) |  | 8.09 (4.26-16.64) | 0.003 |
| Crizotinib | 29 (13.28-62.52) |  | 22.72 (8.93-47.4) | 0.02 |
| PD173074 | 63.7 (42.58-96.26) |  | 52.95 (32.79-85.16) | 0.01 |
| Savolitinib | 13.96 (10.17-19.72) |  | 12.61 (8.58-18.97) | 0.04 |
| Staurosporine | 0.08 (0.04-0.12) |  | 0.05 (0.02-0.1) | 0.001 |
| **WNT signaling** |  |  |  |  |
| IWP-2 | 17.48 (12.41-23.87) |  | 14.48 (10.53-21.87) | 0.01 |

**Abbreviation:** IC50: half maximal inhibitory concentration.
