# Supplementary material for: Identification and external validation of a prognostic signature based on myeloid-derived suppressor cell-related lncRNAs for hepatocellular carcinoma
Source: Hereditas. 2026 Mar 19;163:54. doi: 10.1186/s41065-026-00664-z (PMC13123200; doi:10.1186/s41065-026-00664-z)
Supplement: Supplementary file 7 — Supplementary Material 7. [file 41065_2026_664_MOESM7_ESM.docx]

**Table S7** Antineoplastic drug sensitivity information (no obviously sensitive group).

| **Target pathway** | **Low-risk group** |  | **High-risk group** | **P-value** |
| --- | --- | --- | --- | --- |
|  | **IC50 (25%-75%)** |  | **IC50 (25%-75%)** |  |
| **Apoptosis regulation** |  |  |  |  |
| AZD5582 | 9.34 (3.18-26.96) |  | 9.88 (1.21-45.11) | 0.6622 |
| LCL161 | 156.73 (100.89-213.27) |  | 142.77 (99.26-206.74) | 0.287 |
| MIM1 | 45.46 (24.41-95.81) |  | 53.01 (20.63-131.15) | 0.4408 |
| UMI-77 | 11.78 (5.72-28.28) |  | 8.75 (3.95-44.33) | 0.3985 |
| Venetoclax | 8.78 (5.77-13.2) |  | 8.52 (5.11-15.16) | 0.9374 |
| WEHI-539 | 45.8 (14.76-108.55) |  | 36.2 (9.16-103.85) | 0.2005 |
| **Cell cycle** |  |  |  |  |
| AZD5438 | 4.16 (1.74-14.99) |  | 6.34 (1.95-37.13) | 0.1169 |
| BI-2536 | 1.15 (0.37-3.14) |  | 0.97 (0.35-2.79) | 0.5285 |
| Dinaciclib | 0.03 (0.02-0.09) |  | 0.03 (0.01-0.14) | 0.5947 |
| MK-1775 | 1.89 (1.02-3.95) |  | 1.64 (0.64-5.12) | 0.1625 |
| RO-3306 | 20.92 (13.3-29.03) |  | 22.88 (13.38-35.16) | 0.0776 |
| **Chromatin histone acetylation** |  |  |  |  |
| Vorinostat | 3.87 (2.69-6.2) |  | 4.4 (2.71-6.74) | 0.4891 |
| EPZ004777 | 171.68 (107.57-285.12) |  | 159.98 (89.91-340.93) | 0.8517 |
| EPZ5676 | 244.37 (181.67-326.01) |  | 255.3 (184.35-394.86) | 0.1932 |
| GSK343 | 16.09 (11.49-22.56) |  | 17 (11.02-26.87) | 0.542 |
| **Chromatin other** |  |  |  |  |
| RVX-208 | 114.71 (77.36-180.84) |  | 120.41 (74.9-184.23) | 0.8128 |
| **DNA replication** |  |  |  |  |
| Cyclophosphamide | 197.4 (122.18-332.49) |  | 173.46 (87.09-301.35) | 0.0821 |
| Pyridostatin | 29.18 (17.38-54.06) |  | 28.09 (15.36-49.39) | 0.6629 |
| Temozolomide | 437.11 (274.85-694.98) |  | 395 (228.06-644.41) | 0.1809 |
| **EGFR signaling** |  |  |  |  |
| Erlotinib | 15.87 (9.61-22.62) |  | 14.67 (7.07-26.21) | 0.3667 |
| **ERK MAPK signaling** |  |  |  |  |
| SCH772984 | 13.72 (5.05-36.26) |  | 12.84 (5.12-35.4) | 0.7543 |
| Trametinib | 1.74 (0.45-5.48) |  | 2.64 (0.67-5.98) | 0.1177 |
| Ulixertinib | 18.8 (8.23-31.65) |  | 19.47 (9.8-33.42) | 0.8257 |
| **Genome integrity** |  |  |  |  |
| AZD6738 | 7.78 (3.92-14.88) |  | 7.24 (2.8-22.89) | 0.8083 |
| BIBR-1532 | 137.61 (71.34-260.99) |  | 138.39 (63.12-275.13) | 0.6154 |
| KU-55933 | 70.62 (36.5-146.6) |  | 82.55 (46.87-163.21) | 0.1786 |
| Telomerase Inhibitor IX | 1.48 (0.91-2.98) |  | 1.62 (0.61-5.65) | 0.6693 |
| VE-822 | 24.01 (11.63-54.21) |  | 28.08 (12.37-72.47) | 0.3787 |
| VE821 | 72.85 (38.08-123.87) |  | 60.04 (22.77-126.23) | 0.1578 |
| **IGF1R signaling** |  |  |  |  |
| NVP-ADW742 | 16.56 (10.78-25.23) |  | 15.74 (8.24-26.26) | 0.2559 |
| **Metabolism** |  |  |  |  |
| AGI-5198 | 109.18 (78.93-159.76) |  | 107.57 (65.86-174.29) | 0.727 |
| **Mitosis** |  |  |  |  |
| Vinblastine | 0.02 (0.01-0.06) |  | 0.03 (0.01-0.08) | 0.9809 |
| **Other** |  |  |  |  |
| Dactinomycin | 0.07 (0.03-0.19) |  | 0.1 (0.02-0.28) | 0.3374 |
| IAP_5620 | 160.69 (94.98-263.88) |  | 168.09 (105.5-308.9) | 0.2217 |
| VSP34_8731 | 7.81 (3.87-19.49) |  | 10.15 (4.2-33.64) | 0.0928 |
| Zoledronate | 44.27 (32.72-58.28) |  | 40.76 (29.15-63.31) | 0.4667 |
| **Other, kinases** |  |  |  |  |
| PRT062607 | 28.88 (15.73-45.07) |  | 26.94 (16.76-43.71) | 0.9887 |
| ULK1_4989 | 13.35 (6.38-26.73) |  | 11 (3.6-21.81) | 0.0804 |
| WZ4003 | 38.49 (21.9-71.65) |  | 47.53 (26.22-114.43) | 0.0847 |
| **p53 pathway** |  |  |  |  |
| PRIMA-1MET | 101.19 (49.24-221.66) |  | 89.8 (37.28-281.9) | 0.9801 |
| **PI3K/MTOR signaling** |  |  |  |  |
| Afuresertib | 10.79 (5.68-24.07) |  | 13.02 (6.05-27.18) | 0.2523 |
| AZD8186 | 25.89 (18.58-35.95) |  | 25.87 (17.78-41.38) | 0.6685 |
| Buparlisib | 2.6 (2.17-3.27) |  | 2.68 (2.15-3.37) | 0.7715 |
| CZC24832 | 169.96 (128.25-236.73) |  | 156.78 (106.78-220.22) | 0.0786 |
| MK-2206 | 23.13 (13.68-34.96) |  | 20.19 (11.44-32.01) | 0.1511 |
| OSI-027 | 102.76 (61.97-166.81) |  | 118.13 (65.35-229.61) | 0.093 |
| Uprosertib | 20.81 (11.39-31.5) |  | 20.43 (10.3-40.02) | 0.3835 |
| **Protein stability and degradation** |  |  |  |  |
| Bortezomib | 0.01 (0.01-0.01) |  | 0.01 (0-0.01) | 0.3782 |
| MG-132 | 0.19 (0.15-0.27) |  | 0.2 (0.16-0.29) | 0.2955 |
| P22077 | 80.5 (49.26-127.18) |  | 84.96 (45.31-194.49) | 0.3932 |
| **RTK signaling** |  |  |  |  |
| AZD4547 | 17.95 (10.62-31.29) |  | 15.96 (9.97-30.74) | 0.3631 |
| Foretinib | 2.77 (1.81-3.76) |  | 2.76 (1.82-4.16) | 0.7595 |
| **WNT signaling** |  |  |  |  |
| AZ6102 | 10.22 (7.08-16.11) |  | 11.96 (7-21.98) | 0.0635 |
| MN-64 | 110.57 (58.62-210.29) |  | 98.98 (50.44-248.75) | 0.69 |
| WIKI4 | 43.33 (32.55-60.21) |  | 41.12 (26.37-59.17) | 0.1824 |
| XAV939 | 83.69 (63.75-110.95) |  | 92.13 (53.8-123.24) | 0.9149 |
| **Unclassified** |  |  |  |  |
| Acetalax | 163.61 (58.7-461.41) |  | 150.74 (38.82-343.4) | 0.2232 |
| Gallibiscoquinazole | 11.19 (7-20.43) |  | 13.04 (6.08-30.56) | 0.3959 |
| Carmustine | 422.41 (271-667.75) |  | 450.54 (226.98-807.77) | 0.7514 |
| AZD6482 | 8.82 (1.84-145.92) |  | 14.9 (1.73-315.18) | 0.178 |
| Tozasertib | 15.82 (10.5-28.52) |  | 18.81 (9.92-33.43) | 0.0891 |

**Abbreviation:** IC50: half maximal inhibitory concentration.
