# Supplementary figures and images for: Identification and external validation of a prognostic signature based on myeloid-derived suppressor cell-related lncRNAs for hepatocellular carcinoma
Source: Hereditas. 2026 Mar 19;163:54. doi: 10.1186/s41065-026-00664-z (PMC13123200; doi:10.1186/s41065-026-00664-z)

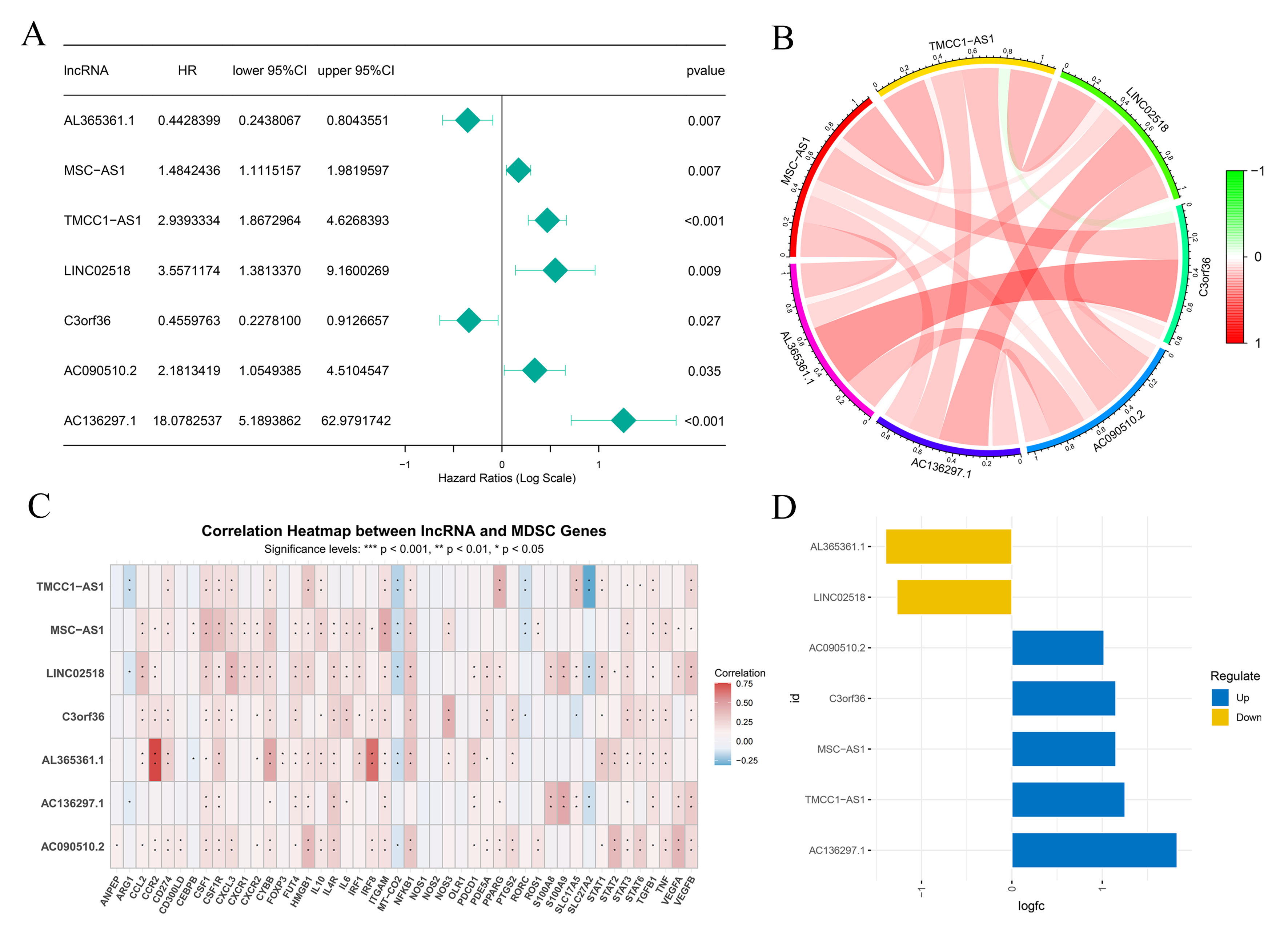

Supplement: Supplementary file 8 — Supplementary Material 8. [file 41065_2026_664_MOESM8_ESM.tif]

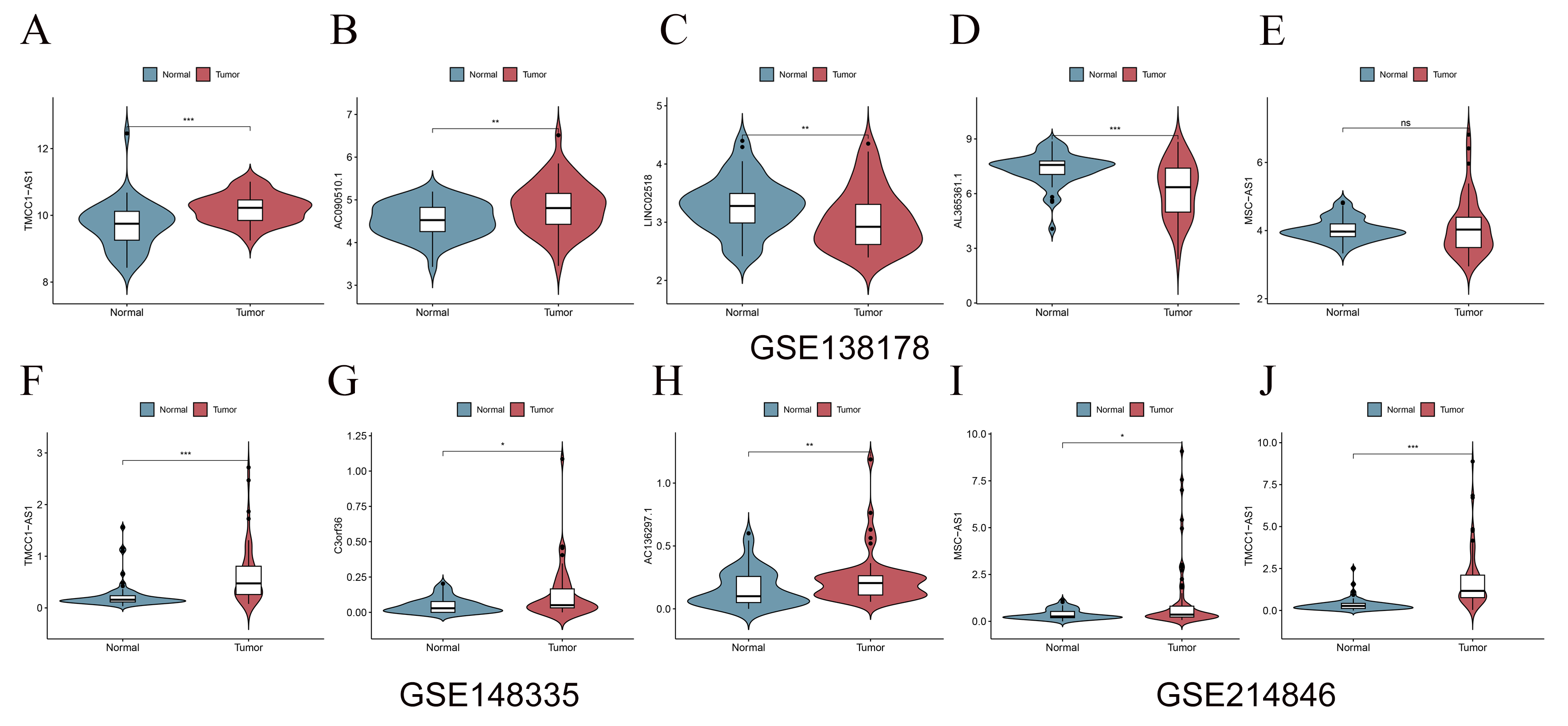

Supplement: Supplementary file 9 — Supplementary Material 9. [file 41065_2026_664_MOESM9_ESM.tif]

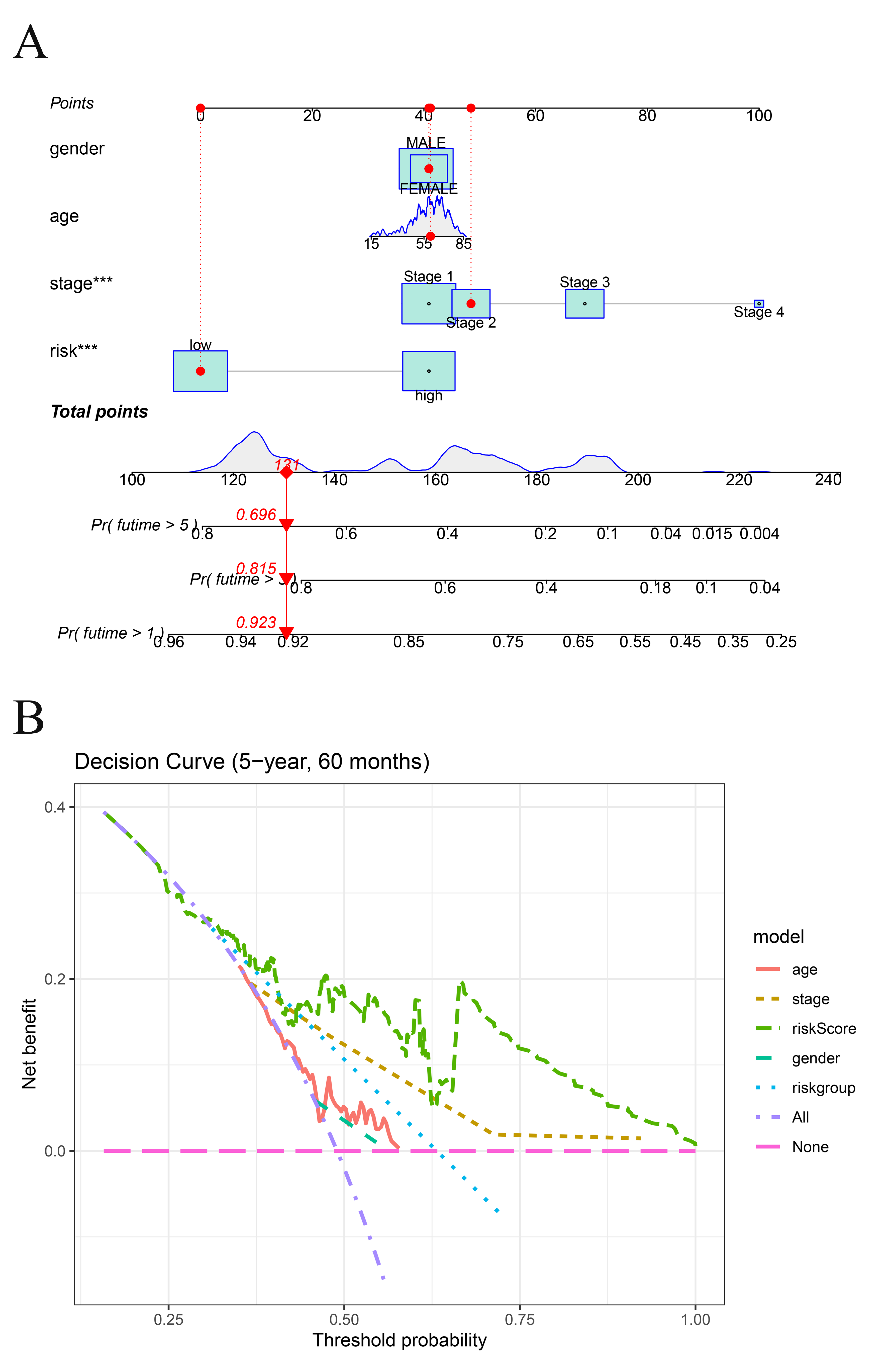

Supplement: Supplementary file 10 — Supplementary Material 10. [file 41065_2026_664_MOESM10_ESM.tif]

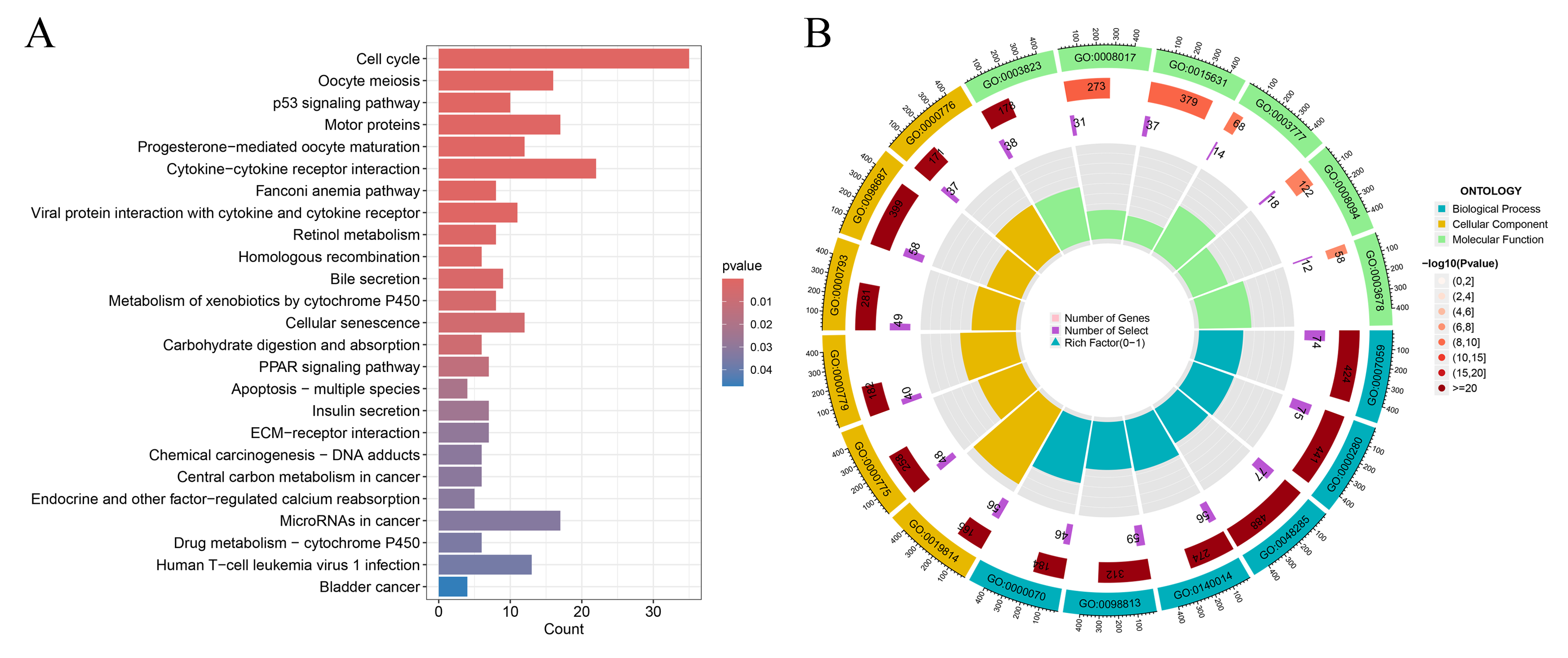

Supplement: Supplementary file 11 — Supplementary Material 11. [file 41065_2026_664_MOESM11_ESM.tif]

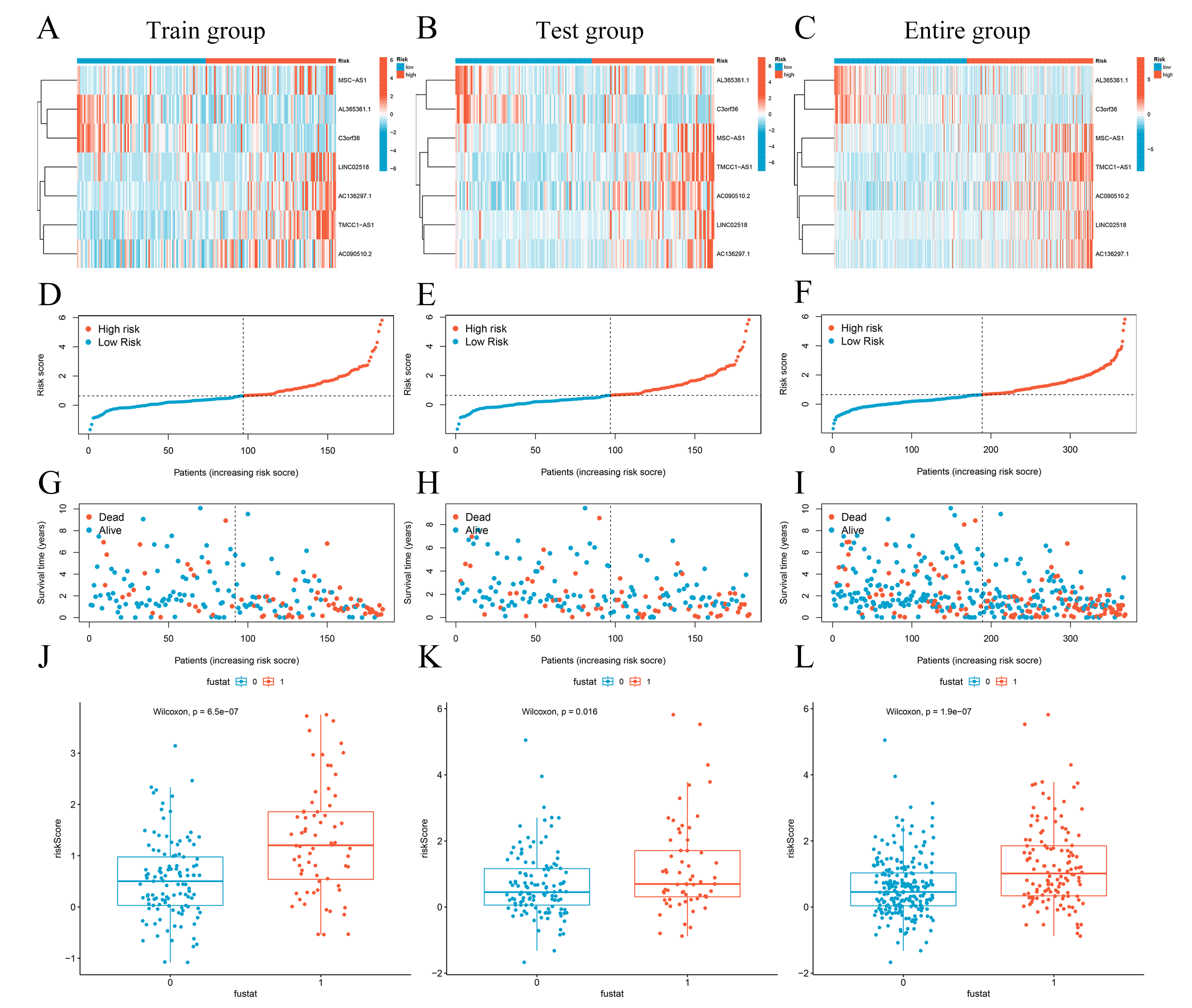

Supplement: Supplementary file 12 — Supplementary Material 12. [file 41065_2026_664_MOESM12_ESM.tif]

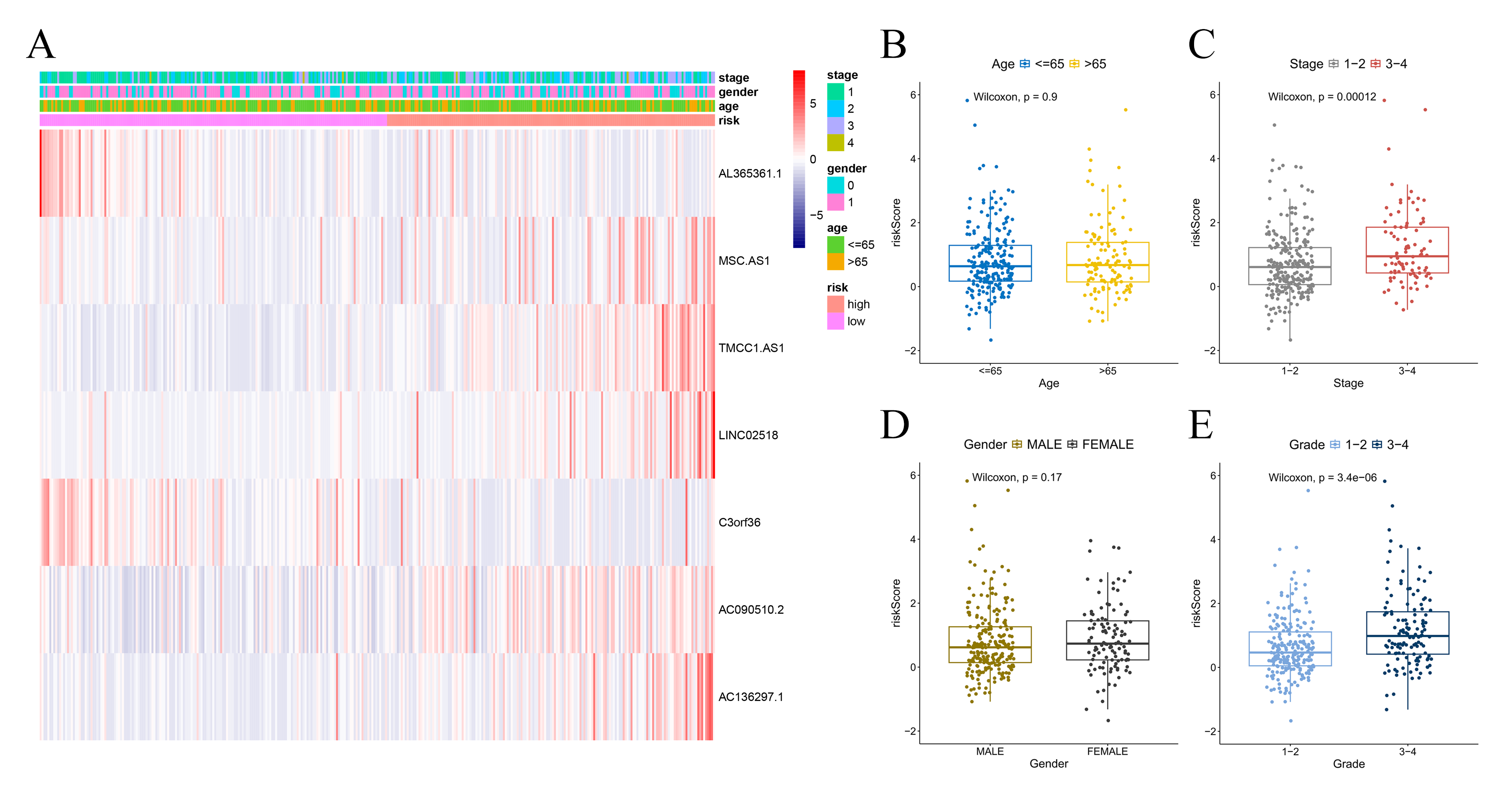

Supplement: Supplementary file 13 — Supplementary Material 13. [file 41065_2026_664_MOESM13_ESM.tif]

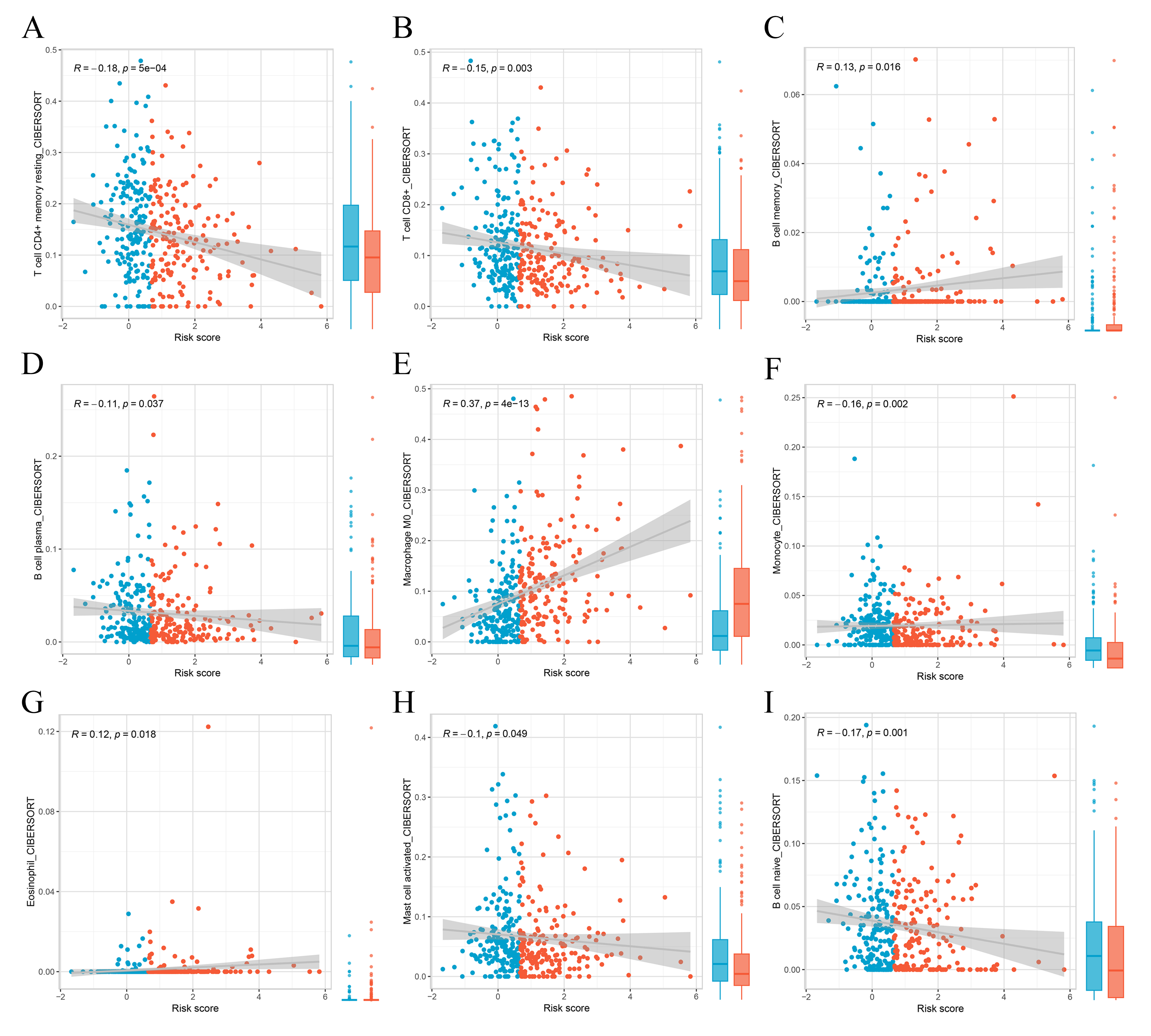

Supplement: Supplementary file 14 — Supplementary Material 14. [file 41065_2026_664_MOESM14_ESM.tif]

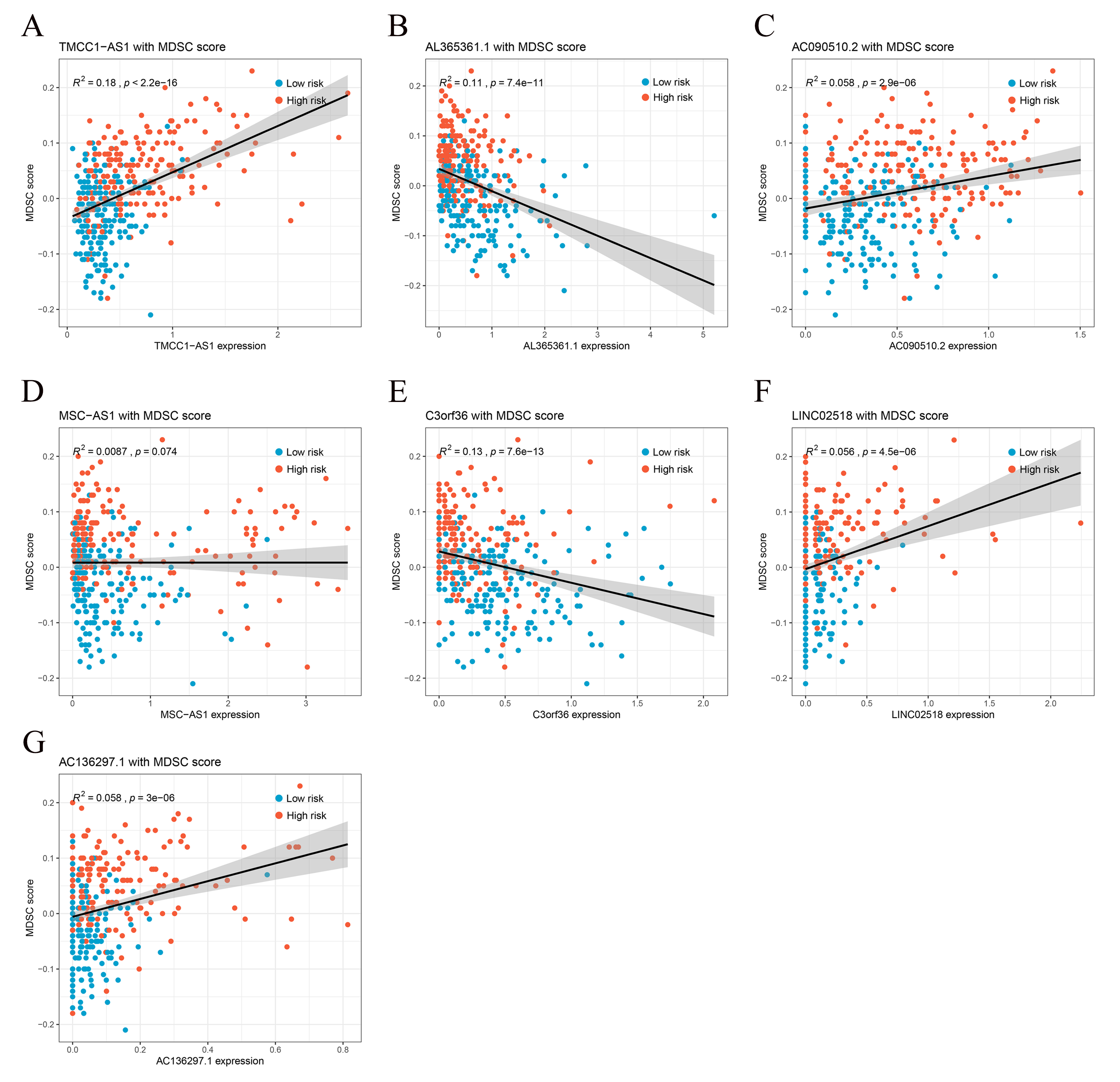

Supplement: Supplementary file 15 — Supplementary Material 15. [file 41065_2026_664_MOESM15_ESM.tif]

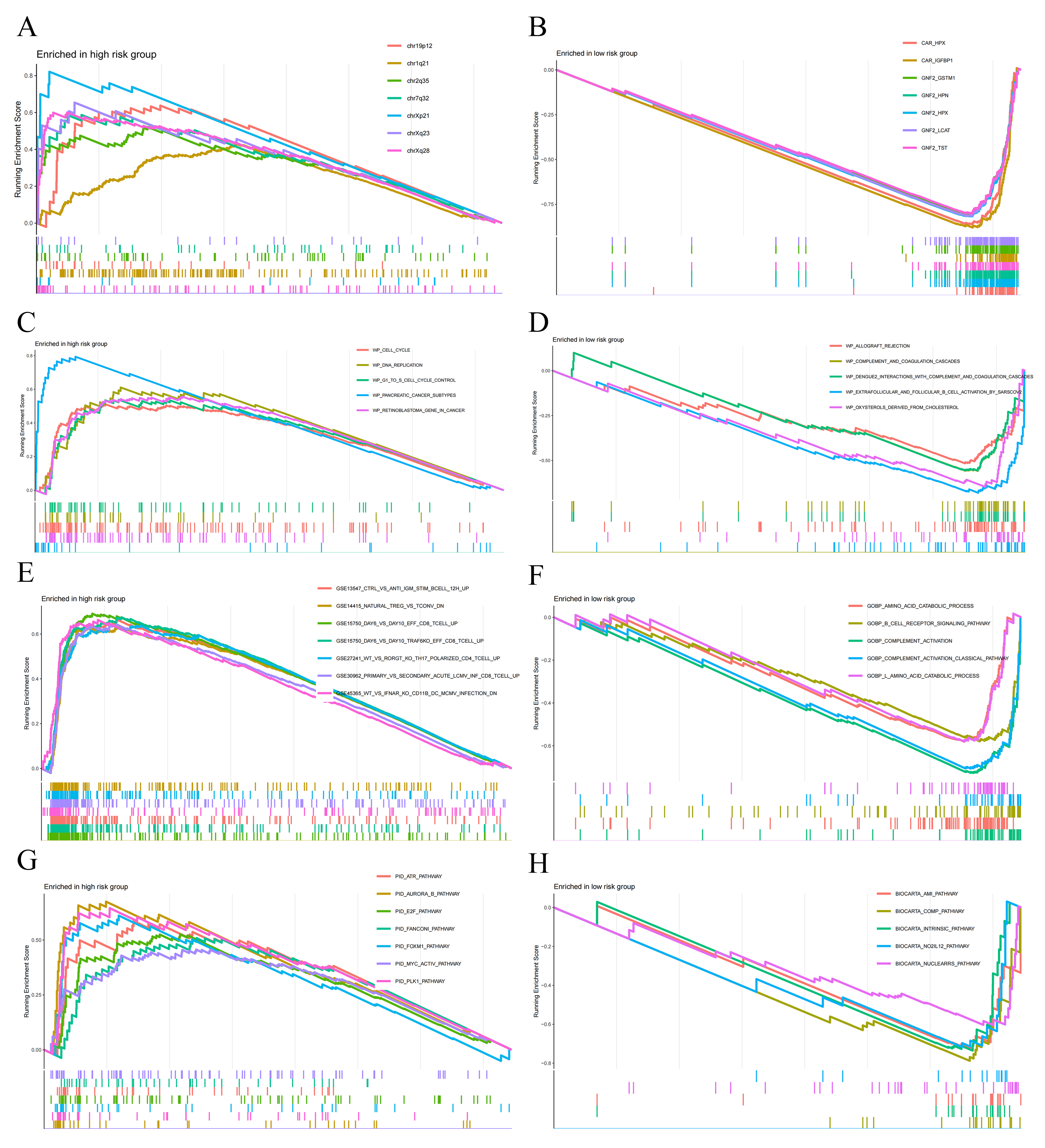

Supplement: Supplementary file 16 — Supplementary Material 16. [file 41065_2026_664_MOESM16_ESM.tif]

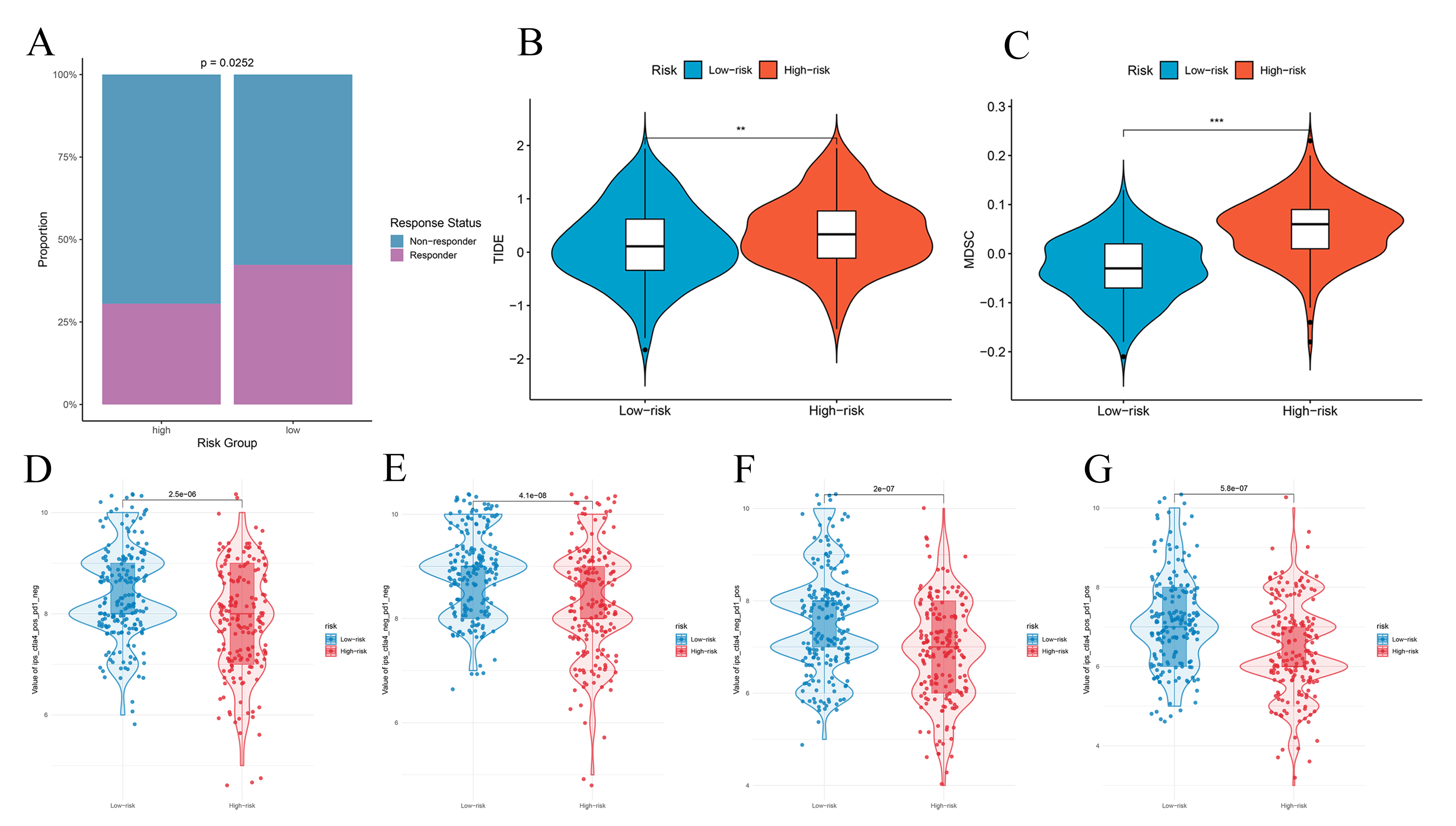

Supplement: Supplementary file 17 — Supplementary Material 17. [file 41065_2026_664_MOESM17_ESM.tif]

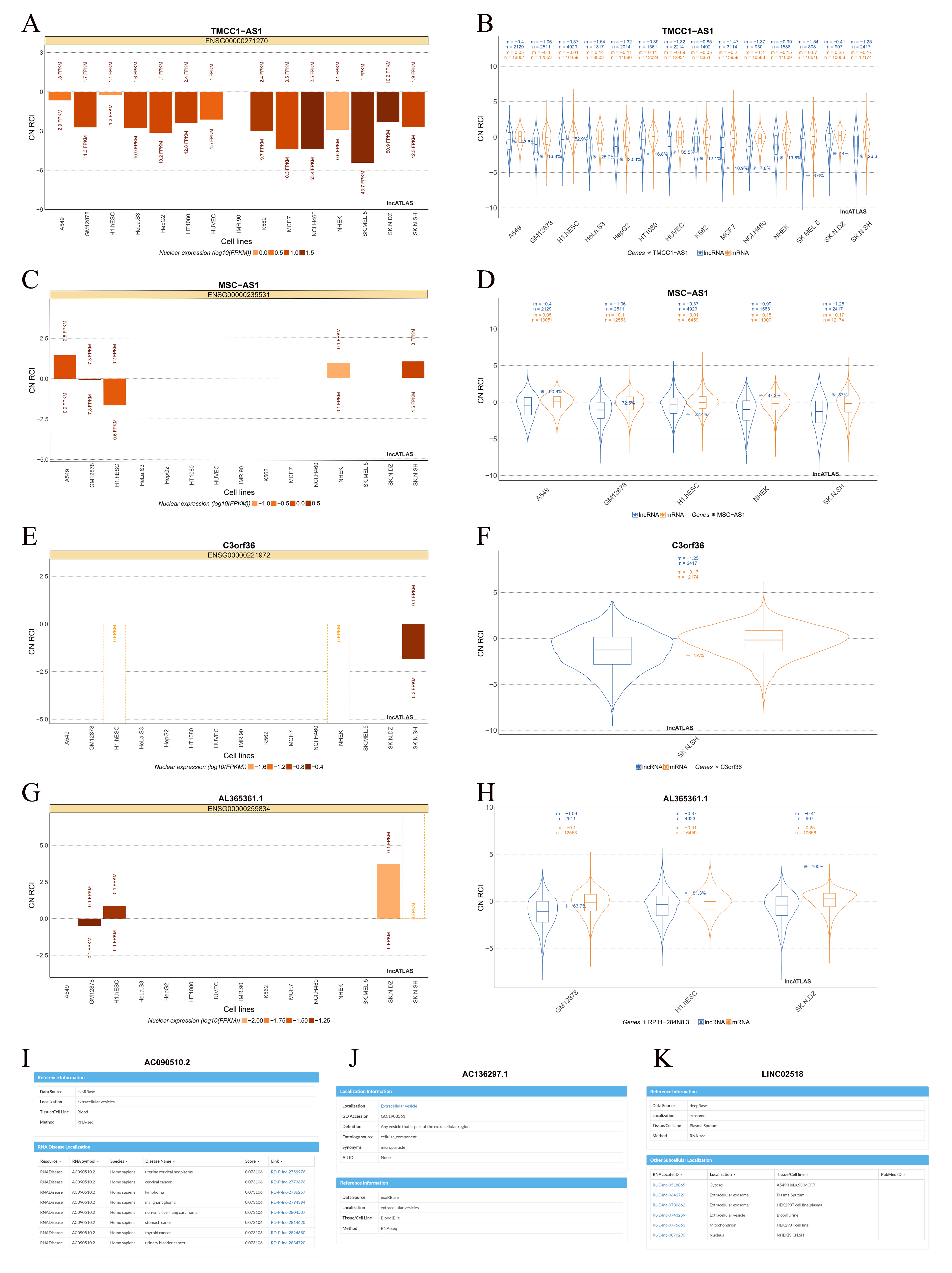

Supplement: Supplementary file 18 — Supplementary Material 18. [file 41065_2026_664_MOESM18_ESM.tif]
